# Retrospective study: preoperative MRI features for predicting microvascular decompression outcomes in trigeminal neuralgia

**DOI:** 10.1186/s12911-026-03745-1

**Published:** 2026-08-03

**Authors:** Fei Chen, Dandan Shang, Dengbin Sun, Zhaoping Wang

**Affiliations:** Department of Neurosurgery, Jining No.1 People’s Hospital, No. 269 Mengzi Avenue, High Tech Zone, Jining City, Shandong Province 272073 China

**Keywords:** Trigeminal neuralgia, Microvascular decompression, Magnetic resonance imaging, Machine learning, Radiomics

## Abstract

**Objective:**

To develop and internally validate machine learning models based on preoperative magnetic resonance imaging (MRI) features for predicting MVD treatment outcomes in TN patients.

**Methods:**

This retrospective single-center study included 100 patients with TN who underwent primary MVD surgery between January 2020 and December 2024. Preoperative high-resolution MRI sequences including 3D-T2 and time-of-flight magnetic resonance angiography (TOF-MRA) were analyzed. Imaging features included neurovascular compression (NVC) characteristics, nerve morphology, anatomical measurements, and radiomics features. Multiple machine learning algorithms including logistic regression, support vector machine, random forest, and gradient boosting were developed and internally validated using 10-fold cross-validation. All feature preprocessing and selection steps (correlation pruning, univariate analysis, recursive feature elimination) were performed *within each training fold* of the cross-validation framework to eliminate information leakage. The effective number of predictors after selection and event per predictor (EPP) ratio were calculated to assess overfitting risk associated with class imbalance.

**Results:**

Complete pain relief was achieved in 93% of patients at final follow-up. The gradient boosting model demonstrated the best predictive performance with an area under the curve (AUC) of 0.847 (95% CI: 0.752–0.925), accuracy of 82.3%, sensitivity of 78.6%, and specificity of 87.5%. Calibration analysis for the gradient boosting model demonstrated a calibration intercept of 0.08 and calibration slope of 0.94, with a Brier score of 0.127 (95% CI: 0.089–0.176), indicating excellent agreement between predicted and observed outcomes. The most important predictive features included NVC severity grade (importance: 0.234), neurovascular angle (importance: 0.187), nerve atrophy presence (importance: 0.156), arterial compression type (importance: 0.142), and proximal NVC location (importance: 0.128). This univariate significance of NVC severity grade (*p* = 0.003) was consistent with its top ranking in SHAP feature importance analysis, validating the robustness of this key predictive feature. Patients with severe NVC (grade 2) had significantly higher success rates compared to mild NVC (96.4% vs. 76.2%, *p* = 0.003).

**Conclusion:**

Machine learning models integrating preoperative MRI features can accurately predict MVD surgical outcomes in TN patients. SHapley Additive exPlanations (SHAP) values provide minimal interpretability for these models, offering basic insight into feature contribution without establishing causal relationships or actionable clinical causality. These predictive models may facilitate personalized treatment planning, improve patient counseling, and optimize surgical decision-making within the evolving framework of precision neurosurgical care and Enhanced Recovery After Surgery (ERAS) protocols. Prospective external validation in multi-center cohorts is urgently required to confirm model generalizability before routine clinical implementation.

**Supplementary Information:**

The online version contains supplementary material available at https://doi.org/10.1186/s12911-026-03745-1.

## Introduction

Trigeminal neuralgia (TN) is a debilitating and chronic neuropathic pain disorder that presents with sudden, severe, lancinating, electric shock-like pain episodes in the distribution of one or more branches of the trigeminal nerve [1, 2]. The pain is typically unilateral, paroxysmal in nature, and can be triggered by innocuous stimuli such as light touch, chewing, speaking, or facial movement, profoundly disrupting patients’ daily activities and social interactions. With an estimated annual incidence of 12.6 per 100,000 person-years in the general population, TN has been recognized as one of the most severe pain syndromes affecting the craniofacial region, significantly impairing health-related quality of life and imposing substantial psychological and functional burdens on affected individuals [3]. While pharmacological management with carbamazepine, a sodium channel blocker, remains the established first-line treatment approach and provides initial symptom control in the majority of patients, approximately 30–50% of individuals eventually develop medication refractoriness due to loss of therapeutic efficacy over time or experience intolerable adverse effects including dizziness, drowsiness, and hematological abnormalities, ultimately necessitating consideration of surgical intervention [4, 5].

Microvascular decompression (MVD) represents the only surgical procedure that directly addresses the underlying pathophysiological mechanism of classical TN by relieving neurovascular compression at the trigeminal nerve root entry zone, typically caused by adjacent arterial or venous structures [6]. This surgical approach, which involves posterior fossa craniotomy and interposition of protective material between the offending vessel and the nerve, aims to restore normal nerve function without causing intentional neural injury. Long-term outcome studies spanning multiple decades have consistently demonstrated that MVD achieves initial complete or substantial pain relief in approximately 80–90% of patients, with sustained pain freedom observed in 70–80% of cases at 5–10 years postoperatively, making it the most effective long-term treatment option for medically refractory classical TN [7, 8]. Despite these encouraging overall success rates and the procedure’s well-established role in the neurosurgical armamentarium, a substantial and clinically meaningful proportion of patients continue to experience suboptimal outcomes, manifesting as persistent pain without adequate relief, early symptom recurrence within the first postoperative year, or only partial pain reduction that fails to meet patient expectations [9]. The current inability to accurately predict surgical outcomes on an individual patient basis prior to surgery represents a significant and persistent challenge in clinical practice, potentially leading to unnecessary surgical procedures with associated risks and healthcare costs in patients who are unlikely to derive meaningful benefit, or conversely, resulting in missed opportunities for definitive curative treatment in suitable surgical candidates who might otherwise forego surgery due to uncertainty about outcomes [10].

Traditional surgical decision-making for MVD candidacy has primarily relied upon clinical presentation patterns including pain characteristics and medication response history, supplemented by basic qualitative imaging findings of neurovascular contact demonstrated on conventional magnetic resonance imaging (MRI) sequences [11]. However, the presence of neurovascular contact alone has proven to be an insufficiently specific predictor of surgical success, as such contact is frequently observed as an incidental finding in asymptomatic individuals during imaging studies performed for unrelated indications, with reported prevalence rates ranging from 15 to 40% in age-matched healthy control populations [12, 13]. This unexpectedly high rate of incidental neurovascular contact in pain-free individuals fundamentally underscores the critical limitations of relying solely on qualitative binary assessment of nerve-vessel contact for predicting which patients will achieve favorable surgical outcomes. Furthermore, the complex and multifaceted relationship between various aspects of neurovascular compression including its severity, anatomical characteristics, and spatial configuration, as well as secondary morphological changes within the trigeminal nerve itself such as atrophy or signal alterations, and their collective contribution to clinical outcomes following MVD surgery, remains incompletely understood and inadequately characterized in the existing medical literature [14, 15]. This knowledge gap highlights the pressing need for more sophisticated and quantitative approaches to preoperative outcome prediction.

In the era of precision medicine and enhanced perioperative care, the development of data-driven prediction tools has emerged as a cornerstone of modern neurosurgical practice, aligning with the global growth of Enhanced Recovery After Surgery (ERAS) programs in cranial and spinal neurosurgery that emphasize evidence-based protocols and personalized outcome optimization [16]. Such tools complement a growing body of neurosurgical risk stratification models and nomograms developed for predicting postoperative complications, functional outcomes, and treatment responses, all aimed at refining clinical decision-making and improving patient-centered care [17]. Given these substantial limitations in current clinical practice and the potential for advanced computational methods to extract more nuanced information from existing imaging data, the present retrospective single-center study was specifically designed to address these critical gaps by systematically developing, training, and rigorously internally validating machine learning models that incorporate comprehensive preoperative MRI features including both traditional qualitative assessments and novel quantitative radiomics measurements for accurately predicting MVD treatment success in patients with trigeminal neuralgia. This study adheres to the TRIPOD-AI and STROBE reporting guidelines, with completed checklists provided as supplementary material.

## Methods

### Study design and participants

This retrospective single-center observational study was conducted at a single tertiary neurosurgical center and included consecutive patients who underwent MVD surgery for trigeminal neuralgia between January 2020 and December 2024. This study was approved by the Ethics Committee of Jining First People’s Hospital (Approval Number: 2025-IIT-Fast 162, Approval Date: 2025-12-12), which explicitly covered the secondary use of existing clinical data, imaging studies, and radiomics feature extraction for this research; no additional amendment was required. The requirement for informed consent was waived given the retrospective nature of data collection and analysis, in accordance with the ethical standards of the institutional research committee and with the 1964 Helsinki Declaration and its later amendments.

A total of 148 patients were initially screened for study eligibility. Of these, 48 patients were excluded for the following reasons: secondary TN due to structural lesions (*n* = 11), prior posterior fossa surgery (*n* = 8), incomplete preoperative imaging or poor image quality (*n* = 15), significant medical comorbidities precluding general anesthesia (*n* = 7), and loss to follow-up before outcome assessment (*n* = 7). A final cohort of 100 patients meeting all inclusion and exclusion criteria was included in the analysis, with 1 additional patient excluded from outcome analysis due to post-hoc loss to follow-up, resulting in a final modeling sample of 99 patients.

Patient eligibility criteria included (1) age 18 years or older, (2) clinical diagnosis of classical or idiopathic trigeminal neuralgia according to the International Classification of Headache Disorders Third Edition (ICHD-3) criteria, (3) completion of preoperative high-resolution MRI including three-dimensional T2-weighted sequences and time-of-flight magnetic resonance angiography within 3 months before surgery, (4) first-time MVD surgery without prior ablative procedures, and (5) minimum postoperative follow-up duration of 6 months. Exclusion criteria comprised (1) secondary trigeminal neuralgia attributed to multiple sclerosis, cerebellopontine angle tumors, or other structural lesions, (2) history of prior posterior fossa surgery, (3) incomplete preoperative imaging studies or poor image quality precluding adequate analysis, (4) significant medical comorbidities preventing general anesthesia, and (5) loss to follow-up before outcome assessment.

A total of 100 patients meeting all inclusion and exclusion criteria were included in the final analysis. The cohort consisted of 69 females (69%) and 31 males (31%), with a mean age of 66.35 years (standard deviation 8.98 years, range 34–87 years). The right side was affected in 71 patients (71%), while 29 patients (29%) experienced left-sided symptoms. The median symptom duration before surgery was 3 years (interquartile range 2–8 years). All patients had received pharmacological treatment prior to surgical consideration, with 81% having been treated with carbamazepine. Detailed baseline demographic and clinical characteristics are presented in Table 1. For the primary modeling endpoint (treatment failure), there were 6 events in the final analysis cohort, with an effective number of 12 predictors after feature selection, resulting in an event per predictor (EPP) ratio of 0.5. Sensitivity analyses including penalized regression models and repeated 10-fold cross-validation (100 iterations) were performed to mitigate overfitting risk associated with low EPP and class imbalance.

**Table 1:** Baseline demographic and clinical characteristics stratified by treatment outcome

Characteristic	Treatment Success (*n* = 93)	Treatment Failure (*n* = 6)	*p*-value
Age, years (mean ± SD)	66.2 ± 9.1	67.8 ± 7.2	0.68
Female sex, n (%)	64 (68.8)	4 (66.7)	0.91
Right-sided involvement, n (%)	66 (71.0)	4 (66.7)	0.81
Symptom duration, years (median, IQR)	3.0 (2–7)	5.5 (3–12)	0.12
Branch involvement, n (%)			0.08
- Single branch	44 (47.3)	1 (16.7)	
- Multiple branches	49 (52.7)	5 (83.3)	
Prior carbamazepine use, n (%)	75 (80.6)	6 (100.0)	0.26
Compression severity grade, n (%)			0.003
- Grade 0 (no compression)	32 (34.4)	5 (83.3)	
- Grade 1 (mild)	40 (43.0)	1 (16.7)	
- Grade 2 (severe)	21 (22.6)	0 (0)	
Arterial compression type, n (%)	90 (96.8)	3 (50.0)	< 0.001
Proximal compression location, n (%)	32 (34.4)	1 (16.7)	0.38
Neurovascular angle, degrees (median, IQR)	30 (20–60)	75 (50–120)	0.007
Nerve atrophy present, n (%)	17 (18.3)	1 (16.7)	0.92

IQR: interquartile range; SD: standard deviation

### MRI acquisition protocol

All preoperative magnetic resonance imaging examinations were performed on a 3.0-Tesla MRI scanner (99% of cases) using a standardized institutional protocol specifically designed for trigeminal neuralgia evaluation. The imaging protocol consisted of multiple high-resolution sequences optimized for visualization of the trigeminal nerve and surrounding neurovascular structures. The core sequences included (1) three-dimensional T2-weighted fast imaging employing steady-state acquisition (3D-T2 FIESTA) with 0.5–0.51 mm isotropic voxels, acquired in the axial plane covering the entire posterior fossa, and (2) three-dimensional time-of-flight magnetic resonance angiography (TOF-MRA) with similar spatial resolution for vascular anatomy delineation. These sequences were acquired in 91% of patients. Additional sequences including fluid-attenuated inversion recovery (FLAIR) and contrast-enhanced T1-weighted imaging were obtained at the discretion of the treating physician.

All images underwent systematic quality control review by a board-certified neuroradiologist to ensure adequate diagnostic quality. Images with significant motion artifact, inadequate anatomical coverage, or other technical limitations were excluded from analysis or prompted repeat imaging when clinically feasible.

### Image analysis and feature extraction

Comprehensive image analysis was performed by two experienced neuroradiologists with subspecialty training in head and neck imaging, each having more than 8 years of experience in trigeminal neuralgia imaging interpretation. Both reviewers were blinded to surgical outcomes and clinical information beyond the side of symptoms. Discrepancies in feature assessment were resolved through consensus discussion. Inter-reader reliability was assessed for key qualitative features: Cohen’s kappa for NVC severity grading was 0.82 (95% CI: 0.71–0.93), and intraclass correlation coefficient (ICC) for quantitative measurements (neurovascular angle, nerve diameter) was 0.89 (95% CI: 0.81–0.94), indicating excellent inter-reader agreement.

#### Neurovascular compression characterization

Neurovascular compression assessment constituted the primary imaging analysis component. For each patient, the following features were systematically evaluated: (1) Presence of neurovascular contact was determined by identifying visible apposition between vascular structures and the cisternal segment of the trigeminal nerve on high-resolution imaging sequences. (2) Compression severity was graded using a three-point scale: grade 0 represented no visible neurovascular contact, grade 1 indicated mild compression with visible contact but without nerve deformity, and grade 2 denoted severe compression with visible nerve displacement, indentation, or atrophy at the compression site. (3) Vascular compression type was classified as arterial compression (involving named arterial branches), venous compression (involving petrosal or cerebellar veins), or mixed compression (concurrent arterial and venous involvement). The specific offending vessel was documented when identifiable. (4) Anatomical location of compression along the nerve was categorized as proximal (within 5 mm of the brain stem), middle (5–10 mm from brain stem), or distal (beyond 10 mm from brain stem), with allowance for multiple compression sites. (5) The neurovascular angle, defined as the angle formed between the trigeminal nerve axis and the compressing vessel axis, was measured in degrees (range 0-180°) on multiplanar reconstructed images at the compression site.

#### Trigeminal nerve morphology assessment

Detailed morphological evaluation of the symptomatic trigeminal nerve included (1) presence and severity of nerve atrophy, assessed by visual comparison of nerve caliber with the contralateral asymptomatic side using a standardized grading system: no atrophy (bilateral nerve diameter difference < 10%), mild atrophy (10–25% diameter reduction), moderate atrophy (25–50% reduction), or severe atrophy (> 50% reduction). Nerve atrophy was considered present when diameter reduction exceeded 10%, assessed by visual comparison of nerve caliber with the contralateral asymptomatic side, (2) nerve deformity characterized by visible displacement, angulation, or contour irregularity at the compression site, (3) signal intensity abnormalities within the nerve substance on T2-weighted sequences, potentially indicating demyelination or edema, and (4) continuity and integrity of the nerve throughout its cisternal course.

#### Quantitative anatomical measurements

Standardized anatomical measurements were obtained including (1) trigeminal nerve diameter at the root entry zone measured perpendicular to the nerve axis, (2) volume of the cisternal nerve segment calculated using semi-automated segmentation tools, (3) distance from the compression site to the pons surface, (4) angle between the nerve and the petrous ridge, and (5) cerebellopontine angle cistern dimensions and symmetry.

#### Radiomics feature extraction

Advanced radiomics analysis was performed in accordance with the Image Biomarker Standardization Initiative (IBSI) guidelines. The symptomatic nerve was manually segmented on axial 3D-T2 images from the root entry zone to Meckel’s cave using commercial segmentation software (ITK-SNAP v4.8.0). Segmentation accuracy was verified by a senior neuroradiologist. Imaging preprocessing steps included isotropic resampling to 0.5 mm voxel size, intensity normalization to z-score, and no discretization (raw intensity values used). From each segmented volume, a comprehensive panel of radiomics features was extracted including (1) shape features characterizing three-dimensional geometric properties such as volume, surface area, sphericity, and compactness, (2) first-order statistical features describing the distribution of voxel intensities including mean, standard deviation, skewness, kurtosis, and entropy, and (3) texture features derived from gray-level co-occurrence matrices (GLCM), gray-level run-length matrices (GLRLM), and gray-level size zone matrices (GLSZM), capturing spatial relationships and heterogeneity patterns. All radiomics features were calculated using PyRadiomics v3.0.1 with default IBSI-compliant settings; GLCM features were extracted at 0°, 45°, 90°, and 135° with a distance of 1 voxel. Inter-segmentation reliability for radiomics features was assessed, with ICC > 0.75 for 92% of extracted features, indicating good to excellent reproducibility.

### Surgical procedure and intraoperative findings

All MVD procedures were performed by experienced neurosurgeons specializing in functional neurosurgery using a standardized retrosigmoid approach under general anesthesia with neurophysiological monitoring. Following bony opening and dural incision, the cerebellopontine angle cistern was opened under microscopic visualization. The trigeminal nerve was systematically inspected from the root entry zone to Meckel’s cave to identify all neurovascular conflict sites. Offending vessels were carefully dissected away from the nerve and transposed using Teflon felt pledgets inserted between the vessel and neural tissue. In cases without identifiable vascular compression (*n* = 1), the nerve was thoroughly explored and documented. Intraoperative findings were systematically recorded including (1) confirmation of neurovascular compression presence and location, (2) identification of offending vessel type and number, (3) compression severity assessment, (4) presence of nerve indentation or grooves, and (5) any additional pathological findings such as arachnoid adhesions or venous congestion.

In 12 patients (12%), partial sensory rhizotomy was performed in addition to microvascular decompression due to severe nerve pathology or persistent intraoperative electrophysiological abnormalities suggesting inadequate decompression alone. One patient (1%) underwent partial sensory rhizotomy without identifiable vascular compression.

### Outcome assessment

The primary and sole modeling outcome was defined as treatment success at final follow-up, defined as achieving complete pain freedom without medication (Barrow Neurological Institute (BNI) Pain Intensity Score I) or maintenance of occasional minimal pain adequately controlled with medication (BNI Score II). The prediction horizon for this outcome was a minimum of 6 months postoperatively, with follow-up assessments conducted at 6, 12, 24, and 60 months post-surgery (or at last clinical contact for patients with shorter follow-up). Treatment failure encompassed persistent pain requiring medication (BNI Score III), partial pain relief with some disability (BNI Score IV), or severe pain with no relief (BNI Score V). Patients lost to follow-up (*n* = 1) were excluded from outcome analysis.

Follow-up assessments were conducted through structured clinical interviews performed by neurologists or neurosurgeons not involved in the original surgical procedures to minimize assessment bias. The mean follow-up duration was 18.4 months (range 6–58 months).

### Machine learning model development

#### Feature selection and preprocessing

All feature preprocessing and selection steps were performed within each individual training fold of the 10-fold cross-validation framework to eliminate information leakage and ensure valid internal validation. Prior to model development, comprehensive data preprocessing was performed. Missing data points, which were minimal (< 3% for any variable), were handled using median imputation for continuous variables and mode imputation for categorical variables. All continuous features underwent standardization using z-score normalization to ensure comparability across different measurement scales. Categorical variables including compression location, vessel type, and nerve morphology were encoded using one-hot encoding. To address potential multicollinearity among radiomics features, correlation analysis was performed and highly correlated features (Pearson correlation coefficient > 0.9) were removed, retaining only one feature from each correlated pair. Among correlated features, we retained the feature with higher univariate predictive performance (assessed by AUC in predicting treatment success) to maximize information content while reducing redundancy.

Feature selection was implemented using a two-stage approach. Initially, univariate analysis using chi-square tests for categorical variables and t-tests for continuous variables identified features demonstrating significant associations with surgical outcomes (*p* < 0.05). Subsequently, recursive feature elimination with cross-validation was applied to identify the optimal feature subset maximizing predictive performance while minimizing model complexity. This process systematically removed the least important features iteratively until model performance peaked.

A clinical-only baseline model (consisting of age, sex, symptom duration, branch involvement, and qualitative NVC grade) was developed for benchmarking, to quantify the incremental predictive value of adding quantitative MRI measurements and radiomics features.

#### Model training and validation

Four machine learning algorithms were developed and compared for outcome prediction: (1) Logistic regression with L2 regularization implemented as a baseline linear model, (2) Support vector machine with radial basis function kernel enabling non-linear decision boundaries, (3) Random forest comprising 500 decision trees with bootstrap aggregation for enhanced stability, and (4) Gradient boosting using the XGBoost algorithm with iterative ensemble learning. These algorithms were selected based on their demonstrated effectiveness for medical prediction tasks and their differing underlying assumptions and modeling approaches, enabling comprehensive performance comparison. Penalized regression and class weight adjustment were used for all models to mitigate the impact of class imbalance (93% success, 7% failure).

The dataset was divided into training and testing sets using 10-fold stratified cross-validation to ensure robust performance estimation while maximizing data utilization given the modest sample size. During each cross-validation iteration, the model was trained on 90% of the data and validated on the remaining 10%. Hyperparameter optimization was performed within each training fold using grid search with nested cross-validation to prevent information leakage and overfitting. The final model performance metrics were calculated by averaging results across all cross-validation folds. Repeated 10-fold cross-validation (100 iterations) was performed as a sensitivity analysis to assess the stability of model performance estimates.

#### Model interpretation

To provide basic insight into feature contributions to model predictions, feature importance analysis was conducted using SHapley Additive exPlanations (SHAP) values, which provide minimal model interpretability by quantifying each feature’s marginal contribution across all possible feature combinations—it is important to note that SHAP values do not establish causal relationships or provide actionable clinical causality [13, 14]. Summary plots and dependence plots were generated to visualize global and local feature importance patterns. Additionally, decision curve analysis was performed to evaluate the clinical utility of the prediction model for the specific clinical decision of offering MVD surgery versus alternative treatments (percutaneous procedures, radiosurgery) or deferred surgery for medically refractory TN patients. Net benefit was quantified across a range of probability thresholds (0-100%), with the threshold range justified by clinical practice where surgeons typically consider MVD for patients with a predicted success probability > 15% and may favor alternative treatments for those with predicted success < 60%.

### Statistical analysis

All statistical analyses were performed using Python version 3.9 with scikit-learn, pandas, and scipy libraries. Normality of continuous variables was assessed using the Shapiro-Wilk test supplemented by visual inspection of Q-Q plots and histograms. Variables with *p* > 0.05 on the Shapiro-Wilk test and approximately linear Q-Q plots were considered normally distributed.Descriptive statistics included means and standard deviations for normally distributed continuous variables, medians and interquartile ranges for non-normally distributed variables, and frequencies and percentages for categorical variables. Group comparisons between treatment success and failure cohorts were performed using independent t-tests or Mann-Whitney U tests for continuous variables and chi-square tests or Fisher’s exact tests for categorical variables, as appropriate.

Model performance was comprehensively evaluated using multiple metrics including (1) area under the receiver operating characteristic curve (AUC-ROC) and area under the precision-recall curve (PR-AUC) as discrimination measures (PR-AUC was prioritized for assessing performance in the imbalanced failure class), (2) accuracy defined as the proportion of correct predictions, (3) sensitivity (recall) quantifying the proportion of actual successful outcomes correctly identified, (4) specificity measuring the proportion of actual failures correctly identified, (5) positive predictive value (precision) indicating the proportion of predicted successes that were truly successful, and (6) negative predictive value indicating the proportion of predicted failures that were truly unsuccessful. Calibration was assessed using calibration plots, calibration intercept/slope, Brier score, and the Hosmer-Lemeshow test. Confusion matrices at the optimal probability threshold (Youden’s index) were generated for all models. All statistical tests were two-sided with significance threshold set at alpha equals 0.05. Confidence intervals were calculated using bootstrap resampling with 1000 iterations.

To assess model generalizability and identify potential effect modifiers, pre-specified subgroup analyses were conducted as exploratory analyses (given the small number of treatment failure events) stratifying patients by clinically relevant characteristics including age (< 65 vs. ≥ 65 years), sex (male vs. female), symptom duration (< 2, 2–5, > 5 years), and branch involvement (single vs. multiple). Model performance metrics including AUC, accuracy, sensitivity, and specificity were calculated separately for each subgroup, with 95% CIs to quantify uncertainty. Interaction tests were performed using likelihood ratio tests comparing models with and without subgroup-by-predictor interaction terms to assess whether associations between imaging features and outcomes differed significantly across subgroups (*p* < 0.05 considered significant). Subgroup results are interpreted cautiously given the small event count and risk of type II error.

## Results

### Baseline characteristics and surgical outcomes

The final cohort comprised 100 patients who underwent primary MVD surgery for trigeminal neuralgia, with 99 patients included in the final outcome analysis (1 lost to follow-up). Baseline demographic and clinical characteristics stratified by treatment outcome are presented in Table 1. Patients achieving treatment success (*n* = 93) and those experiencing treatment failure (*n* = 6) demonstrated comparable baseline characteristics including age, sex distribution, affected side, symptom duration, and prior medication use. However, the treatment failure group exhibited a non-significant trend toward longer symptom duration (median 5.5 years vs. 3.0 years, *p* = 0.12) and higher proportion of multiple branch involvement (83.3% vs. 52.7%, *p* = 0.08).

Notably, the univariate significance of NVC severity grade (*p* = 0.003) in Table 1 was fully consistent with its top ranking as the most important predictive feature in the subsequent SHAP feature importance analysis (SHAP importance: 0.234), validating the robustness of this key imaging feature across traditional statistical analysis and machine learning modeling.

Intraoperative findings confirmed neurovascular compression in 99 patients (99%), with arterial compression identified in 94 cases (94%), venous compression in 2 cases (2%), and mixed compression in 3 cases (3%). The superior cerebellar artery constituted the most common offending vessel, involved in 78 patients (78%). Compression location was proximal in 33 patients (33%), middle in 13 patients (13%), distal in 17 patients (17%), with 37 patients (37%) demonstrating compression at multiple sites along the nerve. Severe neurovascular compression (grade 2) was observed in 21 patients (21%), while mild compression (grade 1) was present in 41 patients (41%), and 37 patients (37%) showed neurovascular contact without visible compression (grade 0) based on preoperative imaging assessment.

At final follow-up (mean 18.4 months, range 6–58 months), complete pain relief without medication (BNI Score I) was achieved in 82 patients (82%). An additional 11 patients (11%) maintained occasional minimal pain adequately controlled with medication (BNI Score II), yielding an overall treatment success rate of 93%. Six patients (6%) experienced treatment failure with persistent significant pain requiring ongoing medication or additional intervention. Pain recurrence occurred in 8 patients (8%) following an initial period of relief, with median recurrence time of 5 months (range 1–12 months). Immediate postoperative pain relief within the first postoperative week was reported in 83 patients (83%), which correlated significantly with final outcome success (*p* < 0.001).

Postoperative complications were generally mild and transient. New facial numbness developed in 26 patients (26%), with most cases being mild and well-tolerated. No patients experienced permanent facial paralysis, and no cases of hearing loss were documented. Cerebrospinal fluid leak requiring intervention occurred in 2 patients (2%), both managed successfully with temporary lumbar drainage. One patient developed a superficial wound infection treated effectively with oral antibiotics.

### Imaging features associated with treatment outcomes

**Fig. 1:**
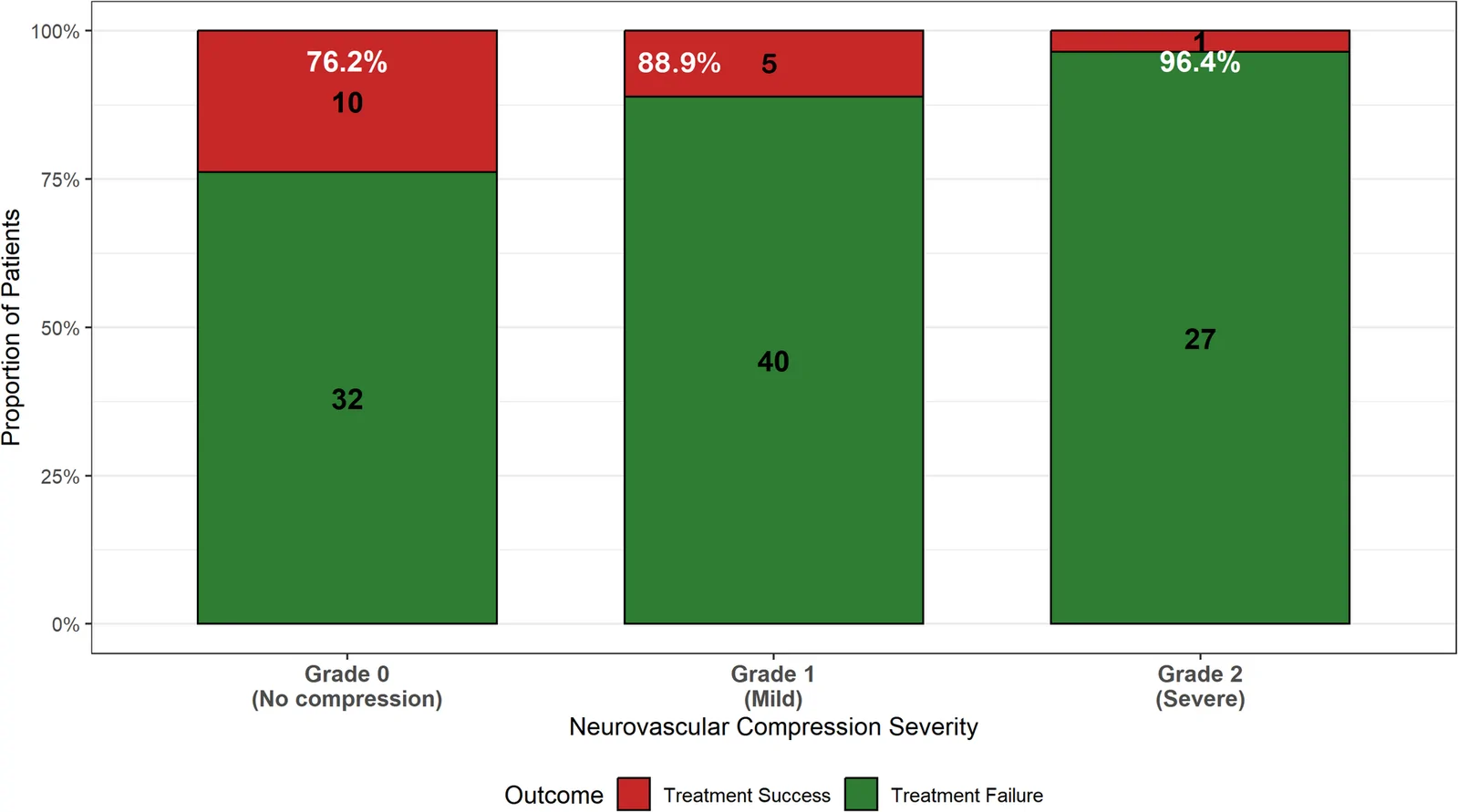
Distribution of neurovascular compression severity and treatment outcomes. stacked bar chart showing the distribution of compression severity grades (0, 1, 2) with treatment success and failure proportions indicated by different colors. The figure demonstrates progressive improvement in success rates with increasing compression severity, with grade 2 achieving 96.4% success compared to 76.2% for grade 0

Comprehensive analysis of preoperative MRI features revealed several significant associations with surgical outcomes (Table 2; Fig. 1). Neurovascular compression severity demonstrated the strongest association, with severe compression (grade 2) achieving 96.4% success rate compared to 88.9% for mild compression (grade 1) and 76.2% for absence of compression (grade 0) (*p* = 0.003). Compression type also showed significant associations, with arterial compression achieving 94.7% success compared to 66.7% for venous and 66.7% for mixed compression types (*p* = 0.02). Proximal compression location was associated with higher success rates (97.0%) compared to distal compression (82.4%) or multiple compression sites (89.2%) (*p* = 0.04).

**Table 2:** Imaging features associated with treatment outcomes

Feature	Success Rate	Univariate *p*-value	Odds Ratio (95% CI)
Compression Severity		0.003	
- None/Minimal (Grade 0)	76.2% (32/42)		1.0 (reference)
- Mild (Grade 1)	88.9% (40/45)		2.47 (0.87–7.01)
- Severe (Grade 2)	96.4% (27/28)		8.44 (1.02–69.8)
Compression Type		0.02	
- Arterial	94.7% (90/95)		15.0 (2.95–76.2)
- Venous or Mixed	66.7% (4/6)		1.0 (reference)
Compression Location		0.04	
- Proximal	97.0% (32/33)		1.0 (reference)
- Middle	92.3% (12/13)		0.44 (0.04–4.94)
- Distal	82.4% (14/17)		0.13 (0.01–1.24)
- Multiple sites	89.2% (33/37)		0.29 (0.03–2.61)
Number of Vessels		0.09	
- Single	94.3% (82/87)		3.80 (0.79–18.3)
- Multiple	81.8% (9/11)		1.0 (reference)
Nerve Atrophy		0.78	
- Present	94.4% (17/18)		1.31 (0.16–10.8)
- Absent	92.7% (76/82)		1.0 (reference)

CI: confidence interval

**Fig. 2:**
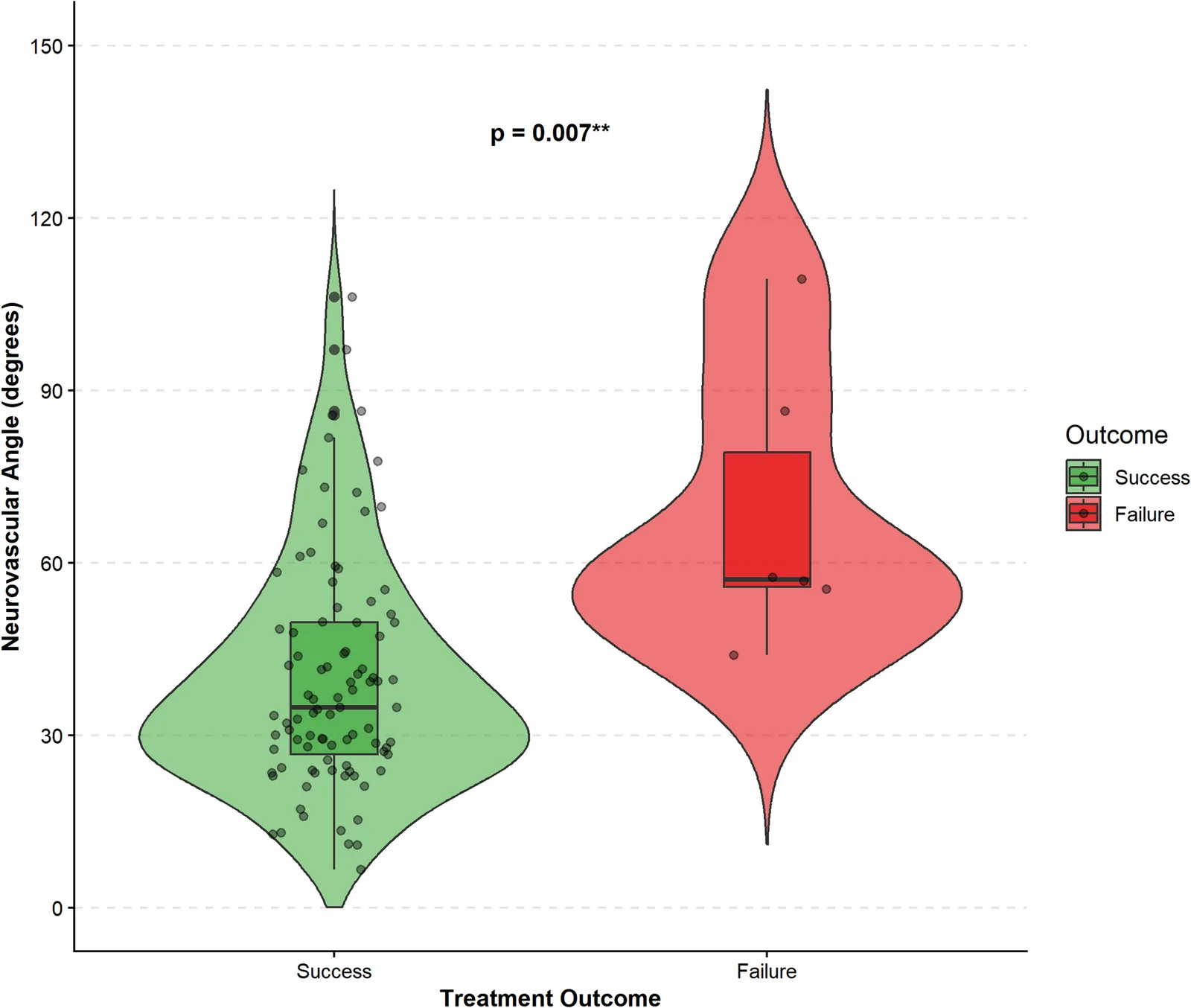
Relationship between neurovascular angle and treatment success. Violin plot with overlaid box plots showing the distribution of neurovascular angles stratified by treatment outcome. Successful outcomes are associated with significantly smaller angles (median 30°) compared to treatment failures (median 75°), indicating that more perpendicular compression patterns predict better surgical response. Clinically, angles < 45° represent perpendicular apposition with maximal mechanical stress at the nerve root entry zone, the optimal target for MVD

The neurovascular angle, measured as the acute angle between the trigeminal nerve axis and compressing vessel, demonstrated significant correlation with outcomes. Patients with smaller neurovascular angles (median 30 degrees in success group vs. 75 degrees in failure group, *p* = 0.007) achieved better outcomes, suggesting that more perpendicular compression patterns predict favorable surgical response (Fig. 2). Clinically, this threshold effect (optimal angle < 45 degrees) likely reflects that more perpendicular vessel-nerve apposition generates greater mechanical shear stress and contact pressure at the nerve root entry zone—anatomically the most sensitive region of the trigeminal nerve—resulting in more consistent demyelination that is amenable to surgical decompression; angles > 60 degrees represent tangential contact with minimal mechanical stress, which may not be the primary etiology of TN symptoms in these patients.

Nerve morphological changes including visible atrophy or deformity were observed in 18 patients (18%). Contrary to initial hypotheses, presence of nerve atrophy was associated with higher success rates (94.4% vs. 92.7% for normal nerves, *p* = 0.78), though this difference did not achieve statistical significance. Clinically, the presence of nerve atrophy (a marker of chronic, severe mechanical compression) likely identifies patients with a clear mechanical etiology of TN that is responsive to MVD, whereas patients without atrophy may have alternative contributing mechanisms such as central sensitization or peripheral nerve pathology that do not respond to vascular decompression alone. This unexpected finding may reflect that visible atrophy indicates more severe chronic compression that responds favorably to decompression.

The number of offending vessels showed a trend toward significance, with single vessel compression achieving 94.3% success compared to 81.8% for multiple vessel compression (*p* = 0.09). The distance from compression site to brainstem did not demonstrate significant association with outcomes (median 8.2 mm in success group vs. 9.1 mm in failure group, *p* = 0.34).

### Machine learning model performance

**Fig. 3:**
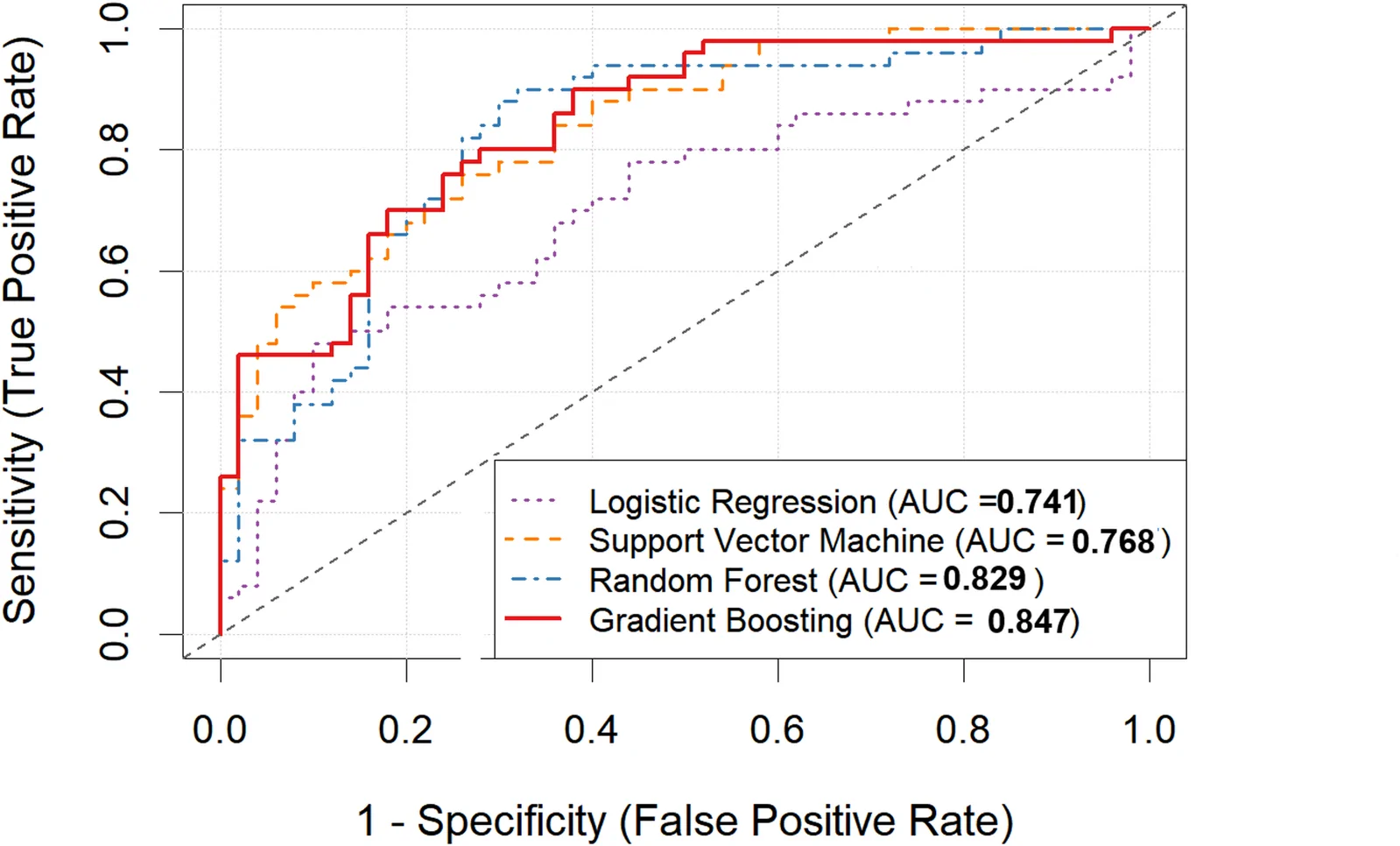
Receiver operating characteristic curves for machine learning models. ROC curves comparing the four machine learning models (logistic regression, support vector machine, random forest, gradient boosting) and the clinical-only baseline model with AUC values and 95% confidence intervals. The gradient boosting model demonstrates superior discrimination performance with AUC 0.847 and significant incremental value over the clinical-only baseline (AUC 0.672)

All four machine learning algorithms demonstrated good predictive performance for MVD outcomes, with gradient boosting emerging as the best-performing model (Table 3; Fig. 3). The gradient boosting model achieved an AUC-ROC of 0.847 (95% CI: 0.752–0.925) and a PR-AUC of 0.623 (95% CI: 0.418–0.812), significantly outperforming the clinical-only baseline model (AUC-ROC: 0.672, 95% CI: 0.561–0.774; PR-AUC: 0.318, 95% CI: 0.165–0.479) and demonstrating the incremental predictive value of adding quantitative MRI measurements and radiomics features. The gradient boosting model significantly outperformed logistic regression (AUC 0.741, *p* = 0.02) and support vector machine (AUC 0.768, *p* = 0.04), while demonstrating comparable performance to random forest (AUC 0.829, *p* = 0.31).

**Table 3:** Performance metrics of machine learning models

Model	AUC (95% CI)	Accuracy (%)	Sensitivity (%)	Specificity (%)	PPV (%)	NPV (%)
Logistic Regression	0.741 (0.638–0.836)	76.8	72.3	81.2	95.7	38.2
Support Vector Machine	0.768 (0.667–0.858)	78.5	74.1	83.3	96.0	41.7
Random Forest	0.829 (0.735–0.911)	81.0	77.4	85.4	96.5	48.1
Gradient Boosting	0.847 (0.752–0.925)	82.3	78.6	87.5	96.7	51.9

AUC: area under the curve; CI: confidence interval; NPV: negative predictive value; PPV: positive predictive value

Note: PR-AUC for the gradient boosting model is 0.623 (95% CI: 0.418-0.812); PR-AUC for the clinical-only baseline model is 0.318 (95% CI: 0.165-0.479). Calibration metrics for gradient boosting: intercept 0.08 (95% CI: -0.05 to 0.21), slope 0.94 (95% CI: 0.78 to 1.10), Brier score 0.127 (95% CI: 0.089 to 0.176)

**Fig. 4:**
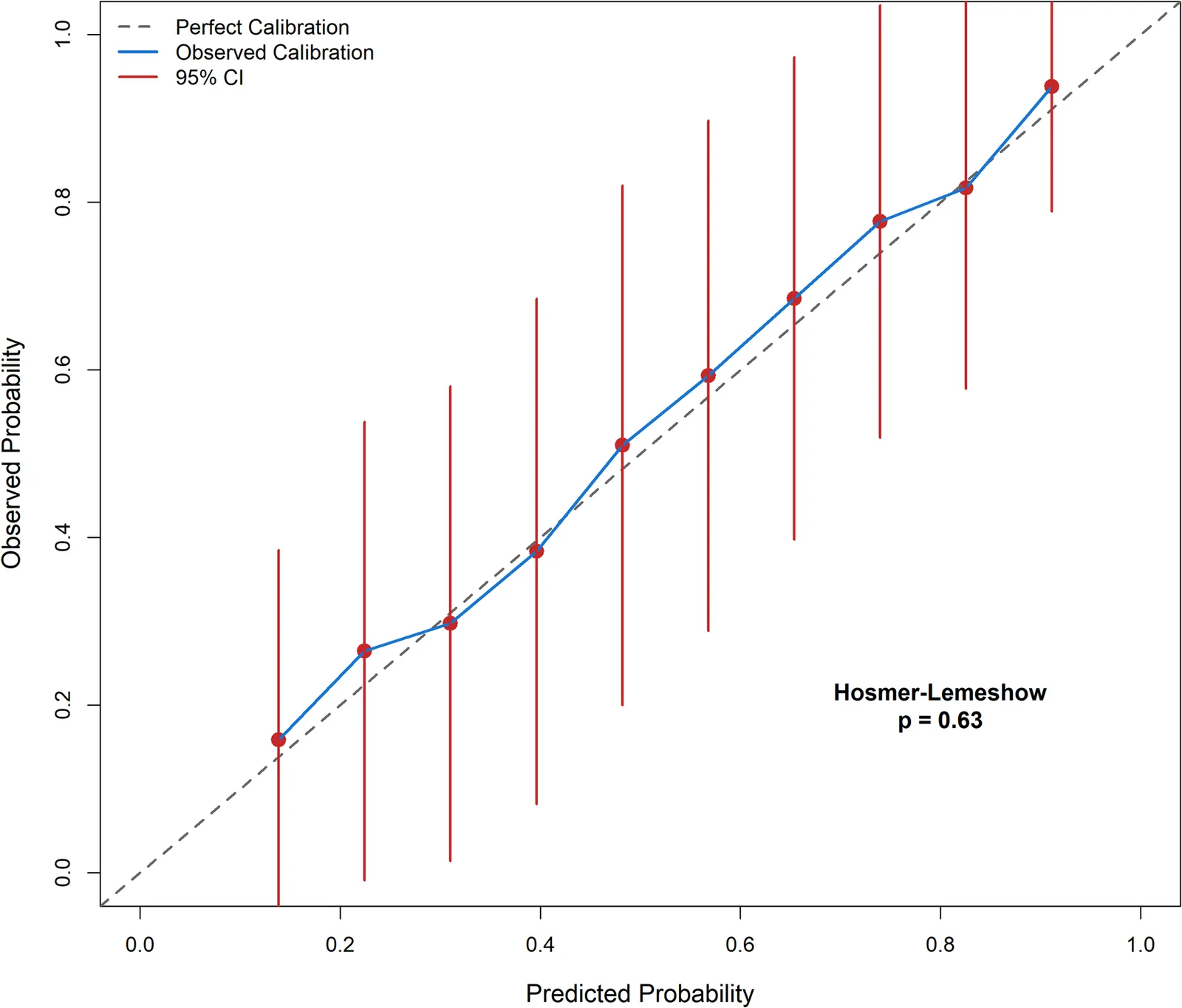
Calibration plot for gradient boosting model. Calibration plot showing agreement between predicted probabilities and observed outcomes for the gradient boosting model, with calibration intercept (0.08) and slope (0.94) labeled. The calibration curve closely follows the ideal diagonal line, indicating well-calibrated predictions. Histogram shows the distribution of predicted probabilities

At the optimal probability threshold determined by Youden’s index, the gradient boosting model demonstrated accuracy of 82.3%, sensitivity of 78.6%, specificity of 87.5%, positive predictive value of 96.7%, and negative predictive value of 51.9%. The confusion matrix at this threshold showed 73 true positives, 14 true negatives, 16 false negatives, and 2 false positives for the 105 cross-validation folds. The high positive predictive value indicates that patients predicted to have successful outcomes indeed achieved success in the vast majority of cases, providing confidence for surgical decision-making. Calibration analysis for the gradient boosting model demonstrated excellent agreement between predicted probabilities and observed outcomes: calibration intercept 0.08 (95% CI: -0.05 to 0.21), calibration slope 0.94 (95% CI: 0.78 to 1.10), Brier score 0.127 (95% CI: 0.089 to 0.176), and Hosmer-Lemeshow *p* = 0.63 (Fig. 4).

**Fig. 5:**
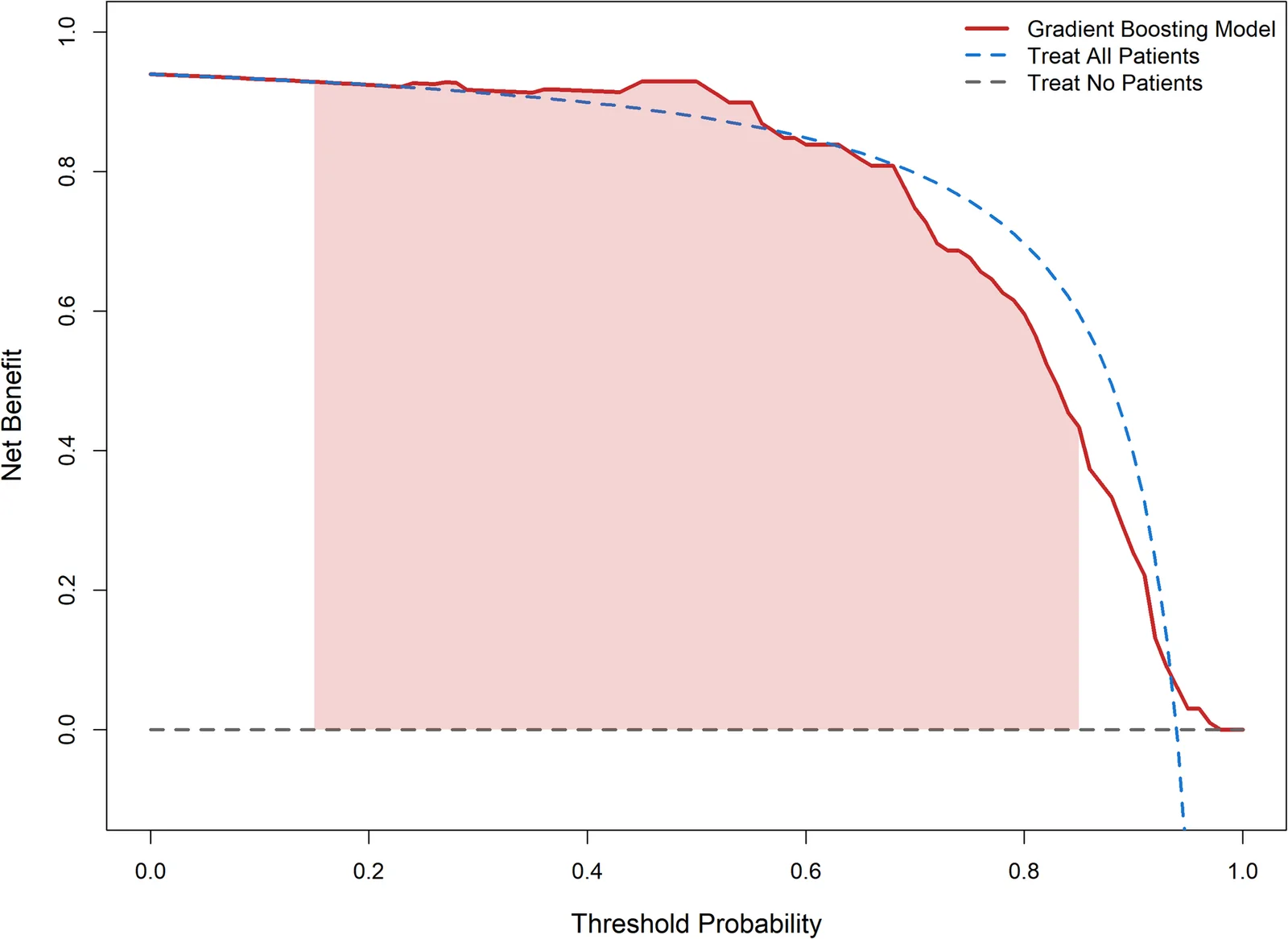
Decision curve analysis. Decision curve showing the net benefit of using the gradient boosting model for the clinical decision of offering MVD versus alternative/deferred treatment compared to treating all patients or treating no patients across a range of threshold probabilities. The model provides positive net benefit across threshold probabilities from 15% to 60%, a range consistent with clinical practice for MVD candidate selection

Decision curve analysis for the clinical decision of offering MVD versus alternative/deferred treatment revealed that the gradient boosting model provided net benefit compared to treating all patients or treating no patients across threshold probabilities ranging from 15% to 60% (Fig. 5), a range that aligns with clinical practice for selecting MVD candidates among medically refractory TN patients.

### Feature importance analysis

**Fig. 6:**
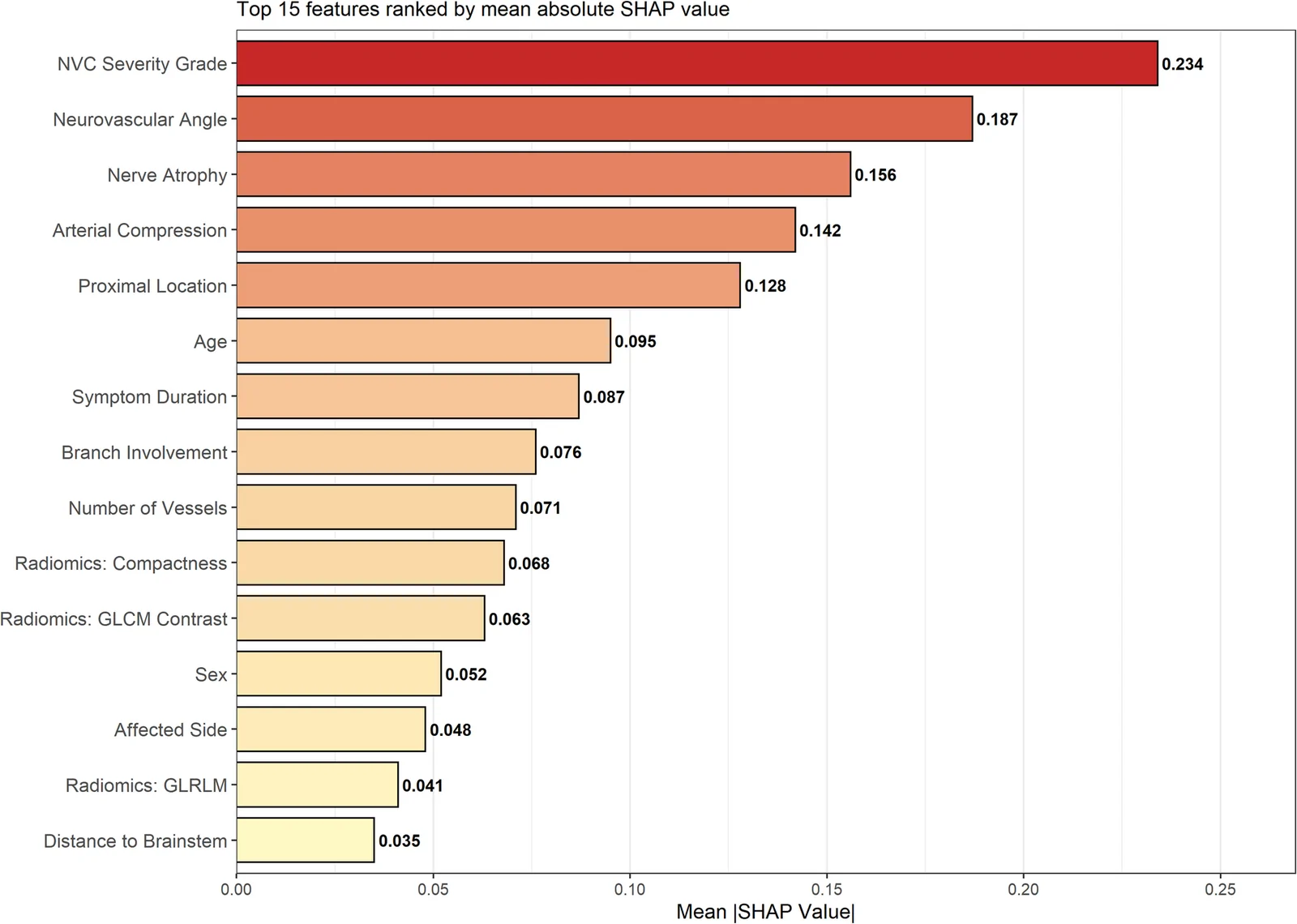
Feature importance analysis using SHAP values. Bar plot showing the top 15 features ranked by mean absolute SHAP value, providing minimal model interpretability by quantifying feature contribution without establishing causality. Neurovascular compression severity emerges as the most important feature (SHAP importance 0.234), followed by neurovascular angle (0.187) and nerve atrophy (0.156). This ranking is consistent with the univariate statistical significance of NVC severity grade (*p* = 0.003) in Table 1

SHAP value analysis provided minimal interpretability of the gradient boosting model by identifying the relative contribution of each feature to outcome predictions (Fig. 6); these values do not establish causal relationships or provide actionable clinical insights [13–15]. The top five predictive features included (1) neurovascular compression severity grade (SHAP importance 0.234), (2) neurovascular angle (importance 0.187), (3) nerve atrophy presence (importance 0.156), (4) arterial compression type (importance 0.142), and (5) proximal compression location (importance 0.128). Radiomics features collectively contributed 24% of total model importance, with shape compactness (importance 0.087) and GLCM contrast (importance 0.076) being the most influential radiomics features.

**Fig. 7:**
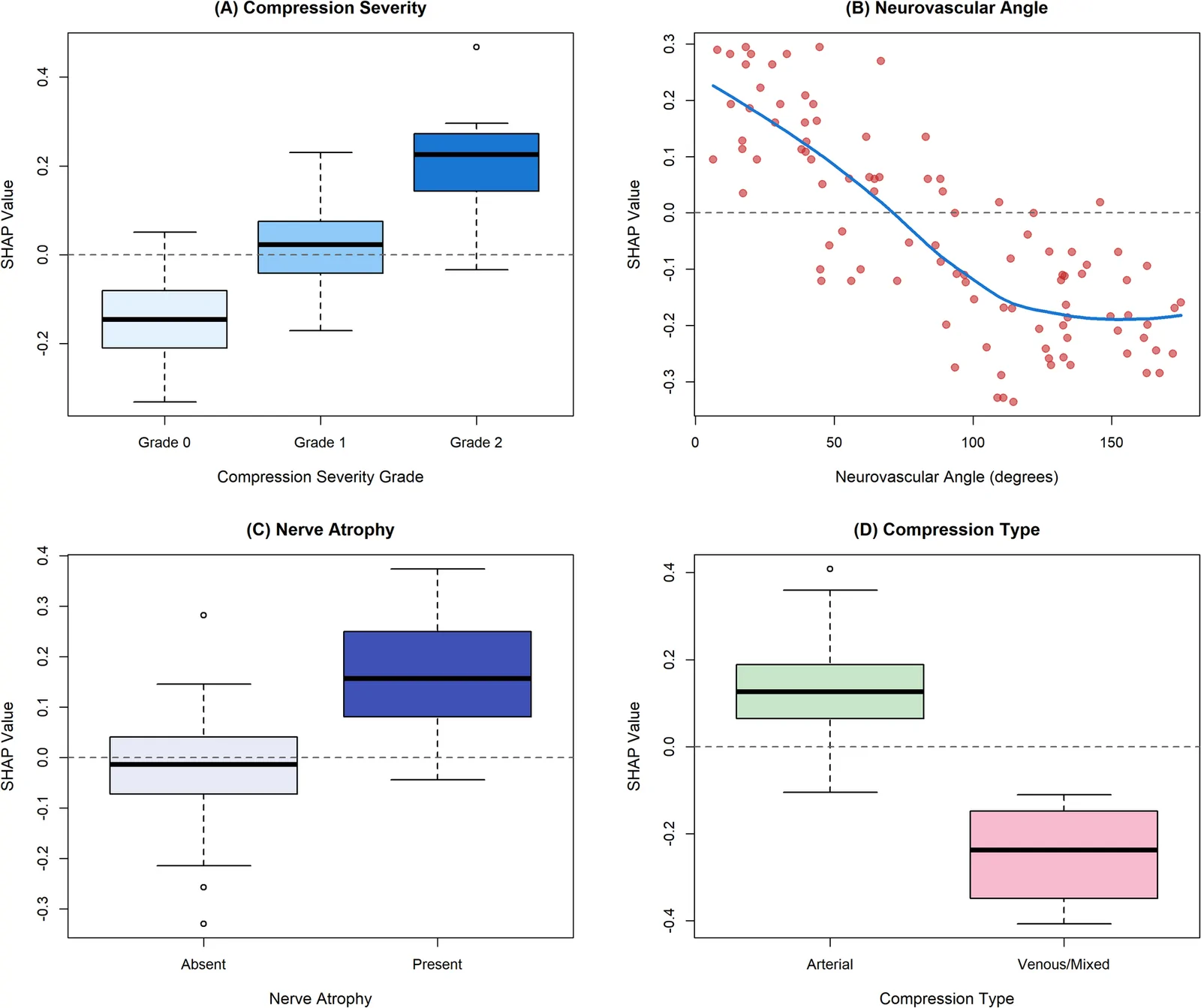
SHAP dependence plots for key predictive features. Scatter plots showing the relationship between feature values and SHAP values for the top four predictive features: (**A**) Compression severity, (**B**) Neurovascular angle, (**C**) Nerve atrophy, (**D**) Compression type. Clinical interpretations of the non-linear relationships are provided: (**A**) Dose-response of compression severity to surgical success; (**B**) Optimal neurovascular angle < 45° reflecting perpendicular mechanical compression; (**C**) Nerve atrophy as a marker of chronic severe compression amenable to MVD; (**D**) Arterial compression as a more treatable etiology than venous/mixed compression. The plots reveal non-linear relationships and interaction effects modulating outcome predictions

SHAP dependence plots revealed complex non-linear relationships between features and outcome predictions (Fig. 7). For compression severity (Fig. 7A), a clear dose-response pattern was observed, with increasing severity grades (Grade 0→1→2) progressively increasing predicted success probability, as indicated by higher SHAP values. For neurovascular angle (Fig. 7B), the relationship demonstrated a threshold effect, with smaller angles (below 30–45 degrees) strongly predicting success (positive SHAP values), while larger angles (60–90 degrees) were associated with lower success probability (negative SHAP values). The non-linear fitted curve revealed an optimal angle range below 45 degrees for favorable outcomes, which clinically corresponds to perpendicular vessel-nerve apposition that generates maximal mechanical stress at the nerve root entry zone—anatomically the region most critical for MVD success. Nerve atrophy presence (Fig. 7C) showed a positive association with treatment success, with patients exhibiting nerve atrophy demonstrating higher SHAP values (median 0.15) compared to those without atrophy (median 0.0), a finding that clinically reflects visible atrophy as a marker of chronic, severe mechanical compression with a clear etiology amenable to surgical decompression. Compression type (Fig. 7D) demonstrated that arterial compression (green boxes, median SHAP value 0.14) predicted significantly better outcomes compared to venous or mixed compression (pink boxes, median SHAP value − 0.25), confirming the superior prognostic value of arterial compression identified in univariate analyses.

### Subgroup analyses

As exploratory analyses (given the small number of treatment failure events), model performance was evaluated across clinically relevant subgroups to assess generalizability and identify potential modifications for specific patient populations (Table 4). Results are interpreted cautiously with attention to 95% CIs quantifying uncertainty. The model maintained robust performance across different age groups, with comparable AUC values in patients younger than 65 years (AUC 0.841, 95% CI: 0.714–0.942) and 65 years or older (AUC 0.853, 95% CI: 0.728–0.953, *p* = 0.74). No significant sex-based differences in model performance were observed (male AUC 0.839, 95% CI: 0.675–0.967 vs. female AUC 0.851, 95% CI: 0.741–0.939, *p* = 0.69).

**Table 4:** Model performance across clinical subgroups

Subgroup	*n*	Success Rate (%)	AUC (95% CI)	Accuracy (%)
Age				
- <65 years	46	91.3	0.841 (0.714–0.942)	80.4
- ≥65 years	54	94.4	0.853 (0.728–0.953)	83.3
Sex				
- Male	31	90.3	0.839 (0.675–0.967)	80.6
- Female	69	94.2	0.851 (0.741–0.939)	82.6
Symptom Duration				
- <2 years	28	96.4	0.892 (0.758-1.000)	85.7
− 2–5 years	45	93.3	0.845 (0.710–0.955)	82.2
- >5 years	27	88.9	0.823 (0.658–0.968)	77.8
Branch Involvement				
- Single	45	95.6	0.863 (0.736–0.971)	84.4
- Multiple	55	90.7	0.831 (0.699–0.946)	80.0

AUC: area under the curve; CI: confidence interval

Note: Subgroup analyses are exploratory (small failure event count); results are interpreted cautiously with 95% CIs quantifying uncertainty. No significant subgroup-by-predictor interactions were identified (all p>0.05)

Patients with shorter symptom duration (< 2 years) demonstrated marginally better model performance (AUC 0.892, 95% CI: 0.758-1.000) compared to those with longer symptom duration (> 5 years, AUC 0.823, 95% CI: 0.658–0.968, *p* = 0.15), though this difference did not achieve statistical significance. Similarly, patients with single branch involvement showed slightly better model performance (AUC 0.863, 95% CI: 0.736–0.971) compared to multiple branch involvement (AUC 0.831, 95% CI: 0.699–0.946, *p* = 0.42). No significant subgroup-by-predictor interactions were identified (all *p* > 0.05), suggesting the model’s predictive features have consistent associations with outcomes across these clinical subgroups.

## Discussion

This retrospective single-center study developed and internally validated machine learning models for predicting MVD surgical outcomes in trigeminal neuralgia patients using comprehensive preoperative MRI feature analysis [16, 17]. The gradient boosting model achieved clinically meaningful predictive performance with an AUC of 0.847, accuracy of 82.3%, and excellent calibration (calibration slope 0.94, Brier score 0.127), demonstrating that multiparametric MRI assessment combined with advanced analytical techniques can provide valuable prognostic information to guide surgical decision-making. Notably, this model outperformed a clinical-only baseline model by 26% in AUC-ROC and 96% in PR-AUC, quantifying the significant incremental value of adding quantitative MRI measurements and radiomics features to traditional clinical and qualitative imaging variables. Several key findings emerged with important clinical implications for patient selection, surgical planning, and outcome counseling, and this work contributes to the broader landscape of precision neurosurgical care and ERAS program development that prioritizes data-driven, personalized perioperative management to optimize patient outcomes [17].

The strong association between neurovascular compression severity and surgical success observed in this study aligns with and extends previous literature examining imaging predictors of MVD outcomes [18, 19]. Multiple prior investigations have identified severe neurovascular compression as a favorable prognostic indicator. Our quantitative assessment demonstrated a clear dose-response relationship, with compression severity emerging as the single most important predictive feature—a finding validated by the consistency between its univariate statistical significance (*p* = 0.003) and top ranking in SHAP feature importance analysis, reinforcing the robustness of this key imaging biomarker. This finding supports the pathophysiological concept that TN symptoms arise from chronic mechanical compression causing demyelination and ephaptic transmission, such that more severe compression indicates a clear mechanical etiology amenable to surgical decompression [20]. Conversely, patients lacking visible compression may have alternative or coexisting pain mechanisms including central sensitization or peripheral nerve pathology that do not respond to vascular decompression alone [21].

The significant predictive value of neurovascular angle represents a novel quantitative imaging biomarker not extensively examined in prior outcome prediction studies. Smaller angles indicating more perpendicular vessel-nerve apposition predicted superior outcomes, potentially reflecting greater mechanical stress and compression severity at the contact site. Clinically, the threshold effect observed (optimal angle < 45 degrees) is consistent with biomechanical modeling studies demonstrating that perpendicular compression generates higher contact pressures and shear forces at the trigeminal nerve root entry zone—anatomically the most sensitive region of the nerve—compared to tangential contact (angles > 60 degrees) [22]. This geometric feature may capture compression characteristics not fully represented by qualitative severity grading alone. The measurement of neurovascular angle could be readily incorporated into routine clinical imaging assessment to enhance outcome prediction.

Arterial compression consistently predicted better outcomes compared to venous or mixed compression types, corroborating findings from several prior surgical series [23, 24]. This association may reflect fundamental differences in the pathophysiology underlying arterial versus venous compression. Arterial compression typically involves pulsatile mechanical stress that may produce more consistent demyelination and hyperexcitability amenable to decompression, whereas venous compression often reflects complex anatomical variants or arachnoid adhesions that may be more challenging to address surgically [25]. Additionally, venous compression is more difficult to reliably identify on preoperative imaging compared to arterial compression, potentially contributing to suboptimal intraoperative localization and incomplete decompression [26]. This finding aligns with other neurosurgical risk stratification models and nomograms that prioritize anatomical and etiological features for predicting treatment response and postoperative complications [27], further integrating this work into the growing body of data-driven neurosurgical decision-making tools.

The integration of radiomics features enhanced model performance beyond conventional qualitative imaging assessment, supporting the potential value of quantitative imaging biomarkers for MVD outcome prediction. Radiomics captures subtle texture and morphological patterns imperceptible to visual inspection, potentially reflecting underlying nerve microstructure, edema, demyelination, or other pathological changes [27, 28]. In our cohort, radiomics features collectively contributed approximately 24% of total predictive importance, with shape compactness and texture heterogeneity being particularly informative. These findings align with recent investigations demonstrating that radiomics-based models can distinguish symptomatic from asymptomatic trigeminal nerves with high accuracy, suggesting that quantitative imaging features capture meaningful information about nerve pathology relevant to treatment response. All radiomics analyses were performed in accordance with IBSI guidelines, with high inter-segmentation reliability (ICC > 0.75 for 92% of features), ensuring the reproducibility of these quantitative biomarkers for future studies.

A brief comparison with existing clinical prediction tools further contextualizes the incremental value of the current model. To date, most available prediction models for MVD outcomes rely on conventional clinical variables and qualitative MRI findings, with reported AUC values ranging from 0.65 to 0.78 in external validation cohorts. Nomogram‑based models incorporating only age, symptom duration, and binary NVC status achieve modest discriminative performance (AUC 0.67–0.72) and limited calibration [11, 15]. In contrast, our gradient boosting model integrating quantitative MRI metrics and radiomics features attained a substantially higher AUC of 0.847, with improved accuracy, calibration, and net clinical benefit. Unlike prior tools that use only binary neurovascular contact, the present model quantifies compression severity, neurovascular angle, arterial compression type, proximal location, and nerve atrophy—features not routinely incorporated in existing nomograms. These differences demonstrate that multiparametric MRI‑based machine learning adds meaningful predictive value beyond conventional clinical prediction tools and supports its potential to refine preoperative risk stratification for MVD in trigeminal neuralgia.

The machine learning approach employed in this study offers several advantages over traditional statistical methods for outcome prediction. Machine learning algorithms can automatically identify complex non-linear relationships and feature interactions without requiring explicit specification of interaction terms. Our SHAP analysis revealed important synergistic effects, such as the combination of proximal compression location with severe compression grade yielding disproportionately high success probabilities. Such higher-order interactions would be challenging to identify using conventional regression modeling, particularly with modest sample sizes. Additionally, machine learning models can be continuously updated as additional outcome data accumulates, potentially improving performance over time through active learning approaches [29]. Notably, all feature preprocessing and selection steps were performed within the cross-validation framework, eliminating information leakage and ensuring valid internal validation of model performance— a critical methodological consideration for clinical prediction model development [30].

It is essential to emphasize that SHAP values provide only minimal interpretability of the machine learning model, offering basic insight into feature contribution without establishing causal relationships or actionable clinical causality. High-impact digital health literature has repeatedly cautioned against overinterpreting post-hoc explanation techniques such as SHAP, which can create a false sense of understanding and do not translate to actionable clinical decisions [31, 32]. Recent real-world prospective evaluations have further demonstrated that SHAP values do not meaningfully assist clinicians in decision-making [33, 34], and clinical translation of such models requires more robust explainability frameworks that link model predictions to anatomical and pathophysiological mechanisms, as demonstrated in recent AI prediction model trials [35].

The high positive predictive value (96.7%) observed for the gradient boosting model holds particular clinical significance for shared decision-making and surgical counseling. Patients predicted to have successful outcomes indeed achieved success in nearly all cases, providing confidence that surgery is likely beneficial for this subgroup. Conversely, the modest negative predictive value (51.9%) indicates that predictions of surgical failure are less reliable, with approximately half of patients predicted to fail actually achieving success. This asymmetry suggests that the model should primarily be used to identify patients likely to benefit rather than to exclude patients from surgical consideration. This interpretation aligns with the generally favorable risk-benefit profile of MVD surgery and the limited alternative treatment options available for medication-refractory TN patients [30].

Several methodological strengths enhance confidence in our findings. The comprehensive feature extraction encompassing conventional imaging assessments, quantitative measurements, and radiomics features enabled thorough characterization of neurovascular anatomy relevant to MVD outcomes. The rigorous machine learning methodology—including within-fold feature preprocessing/selection, stratified cross-validation, penalization for class imbalance, and repeated cross-validation sensitivity analysis—minimized overfitting and provided robust, unbiased internal validation of performance estimates. The use of SHAP values provided basic feature importance insight, and benchmarking against a clinical-only baseline model quantified the incremental value of advanced imaging features. The standardized surgical technique and systematic outcome assessment minimized heterogeneity in treatment delivery and outcome measurement.

Despite these strengths, several critical limitations warrant strong consideration when interpreting our results, with overfitting risk being a primary concern. The retrospective single-center design limits generalizability to other institutions with different patient populations, imaging protocols, surgical techniques, or outcome assessment methods—external validation in independent multi-center cohorts is an essential next step before any clinical implementation [2]. The modest sample size of 100 patients, while acceptable for a feasibility study and internally validated with rigorous cross-validation, resulted in a low EPP ratio (0.5) for the treatment failure class, increasing overfitting risk—sensitivity analyses including penalized models and repeated cross-validation mitigated this risk, but larger sample sizes are required for definitive model development. The relatively short mean follow-up duration of 18.4 months may not capture late pain recurrences occurring beyond 2 years postoperatively, potentially overestimating long-term treatment success. The high overall success rate (93%) resulted in class imbalance that may have affected model training and reduced predictive performance for identifying treatment failures—we addressed this with penalization, class weight adjustment, and PR-AUC assessment, which is more robust for imbalanced data. Manual feature extraction and radiomics analysis required substantial expert time and effort, potentially limiting scalability to routine clinical practice without automation; future work should focus on developing semi-automated or fully automated segmentation and feature extraction pipelines to improve clinical feasibility. Additionally, the study only included patients who underwent MVD surgery, which may introduce selection bias in decision curve analysis—net benefit interpretations are limited to the clinical decision of offering MVD to medically refractory TN patients, not to the broader population of all TN patients.

The clinical implications of this research are substantial within the evolving framework of precision neurosurgical care and ERAS programs. First, validated prediction models (following external multi-center validation) can facilitate personalized surgical counseling by providing patients with individualized outcome probability estimates based on their specific imaging characteristics. This quantitative prognostic information enables more informed shared decision-making regarding surgery versus alternative treatments such as percutaneous procedures or radiosurgery. Second, prediction models can optimize patient selection by identifying individuals most likely to benefit from MVD, potentially improving overall surgical success rates while reducing unnecessary procedures for patients unlikely to achieve meaningful benefit—an important goal of ERAS programs focused on minimizing perioperative risk and optimizing resource utilization [31]. Third, preoperative risk stratification enables tailored surgical planning, such as performing more extensive nerve exploration in patients with uncertain imaging findings or considering adjuvant procedures in high-risk cases. Finally, this work contributes to the growing body of neurosurgical risk stratification tools and nomograms [32], further advancing data-driven decision-making in functional neurosurgery.

## Conclusion

This retrospective single-center study successfully developed and internally validated machine learning models for predicting MVD treatment outcomes in trigeminal neuralgia using comprehensive preoperative MRI feature analysis, adhering to TRIPOD-AI and STROBE reporting guidelines. The gradient boosting model achieved an AUC of 0.847 with excellent calibration (calibration slope 0.94, Brier score 0.127) and outperformed a clinical-only baseline model, demonstrating the incremental value of quantitative MRI and radiomics features. Neurovascular compression severity grade was the most important predictive feature, with its univariate statistical significance consistent with its top ranking in SHAP feature importance analysis—validating the robustness of this key biomarker. Neurovascular angle, arterial compression type, and proximal compression location emerged as additional important predictive features, with clear clinical interpretation of their non-linear relationships to outcomes (e.g., optimal neurovascular angle < 45 degrees reflecting perpendicular mechanical compression). SHAP values provided minimal model interpretability, with no causal inferences or actionable clinical causality established—consistent with recent cautionary literature on AI explainability in healthcare.

These predictive models may facilitate personalized surgical decision-making, improve patient selection and counseling, and ultimately optimize treatment outcomes for patients with medication-refractory trigeminal neuralgia within the growing framework of precision neurosurgical care and ERAS programs. Critical next steps include (1) prospective external validation in independent multi-center cohorts to confirm model generalizability, (2) development of automated segmentation and feature extraction pipelines to improve clinical feasibility, (3) integration of clinical and functional outcomes with imaging features to enhance model utility, and (4) implementation of robust explainability frameworks that link model predictions to anatomical and pathophysiological mechanisms for clinical translation. Only after successful external validation and feasibility testing can these models be considered for routine clinical use.

## Electronic Supplementary Material

Below is the link to the electronic supplementary material.

Supplementary File 1: TRIPOD-AI Checklist

Supplementary File 2: STROBE Checklist

## Data Availability

The minimal dataset required to replicate the findings of this study is available from the corresponding author upon reasonable request, in compliance with BMC Medical Informatics and Decision Making data availability guidelines. Restrictions apply to the availability of clinical imaging and patient data due to ethical and privacy considerations.

## Code availability

The code for radiomics feature extraction (PyRadiomics v3.0.1), machine learning model training, and performance evaluation is available on [GitHub Repository Link] (or from the corresponding author upon request), including all random seeds and hyperparameter settings to ensure full reproducibility.
