# Supplementary material for: Retrospective study: preoperative MRI features for predicting microvascular decompression outcomes in trigeminal neuralgia
Source: BMC Med Inform Decis Mak. 2026 Aug 3;26:443. doi: 10.1186/s12911-026-03745-1 (PMC13721266; doi:10.1186/s12911-026-03745-1)
Supplement: Supplementary file 1 [file 12911_2026_3745_MOESM1_ESM.docx]

**TRIPOD-AI Checklist (2024) for Clinical Prediction Models Using Machine Learning**

**Study Title**: Feasibility of Preoperative MRI Features for Predicting Microvascular Decompression Outcomes in Patients with Trigeminal Neuralgia

**Reporting Status**: Y=Reported in manuscript; N=Not reported; NA=Not applicable

| **Domain** | **Item No.** | **Checklist Item** | **Report Status** | **Manuscript Location** | **Key Details/Remarks** |
| --- | --- | --- | --- | --- | --- |
| **Title & Abstract** | 1 | State if the study develops, validates, updates, or extends a prediction model; specify the study design (e.g., cohort, case-control) | Y | Title/Abstract (full) | Retrospective single-center cohort study; **model development + internal validation** for MVD outcome prediction |
| **Title & Abstract** | 2 | Specify the study population, predictors, outcome(s), prediction time horizon, and key model performance measures | Y | Abstract (Objective/Methods/Results) | Population: TN patients undergoing primary MVD; Predictors: Preoperative MRI features; Outcome: MVD treatment success; Time horizon: ≥6 months post-op; Key metric: XGBoost AUC=0.847 (95%CI:0.752-0.925) |
| **Introduction** | 3 | Explain the clinical need for the prediction model and its potential intended use(s) | Y | Introduction (1-4) | Address unmet need for preoperative MVD outcome prediction; Intended use: Surgical decision-making, patient counseling, personalized treatment planning |
| **Introduction** | 4 | State the research question(s) (e.g., which predictors to include; how well the model predicts the outcome; if the model adds value to existing tools) | Y | Introduction (4) | Develop/validate MRI-based ML models for MVD outcome prediction; quantify incremental value over clinical-only baseline models |
| **Methods: Study Design** | 5 | Describe the study design (e.g., cohort, case-control) and data source(s); specify the time period of data collection | Y | Methods (2.1) | Retrospective single-center cohort; Tertiary neurosurgical center; Data collection: Jan 2020–Dec 2024 |
| **Methods: Study Design** | 6 | Describe the model development and validation process (e.g., development cohort, internal/external validation; if multiple imputation or cross-validation is used) | Y | Methods (2.6)/Discussion (8) | Development + **10-fold stratified cross-validation (internal validation)**; no external validation (planned as future work); missing data imputation (median/mode) |
| **Methods: Study Design** | 7 | Describe how the sample size was determined (e.g., formal calculation, feasibility; justify the number of events per predictor [EPP/EPV]) | Y | Methods (2.1) | Feasibility study (n=100); EPP=0.5 (6 failure events, 12 final predictors); sensitivity analyses to mitigate overfitting |
| **Methods: Study Population** | 8 | Define the study population with explicit eligibility (inclusion/exclusion) criteria; specify the setting (e.g., primary/secondary care) | Y | Methods (2.1) | Inclusion: ≥18y, ICHD-3 classical TN, primary MVD, pre-op high-res MRI, ≥6m follow-up; Exclusion: Secondary TN, prior surgery, poor imaging, loss to follow-up; Setting: Tertiary neurosurgical center |
| **Methods: Study Population** | 9 | Report the flow of participants through the study (e.g., number screened, eligible, included, excluded [with reasons], and analyzed for the outcome) | Y | Methods (2.1)/SM | 148 screened → 48 excluded → 100 included → 99 analyzed (1 lost to follow-up); **CONSORT-style flow diagram (SM)** |
| **Methods: Study Population** | 10 | Describe the follow-up procedures (e.g., duration, frequency, assessment methods) for outcome ascertainment | Y | Methods (2.5)/Results (3.1) | Follow-up: Mean 18.4 months (range 6-58); Structured clinical interviews by independent neurologists/neurosurgeons; Outcome assessed at final follow-up |
| **Methods: Predictors** | 11 | List all predictors considered (including clinical, biological, imaging, and omics features); specify the timing of predictor assessment (relative to the outcome) | Y | Methods (2.3) | 4 categories: NVC characteristics, nerve morphology, anatomical measurements, radiomics features; All assessed **preoperatively (≤3m before MVD)** |
| **Methods: Predictors** | 12 | Describe how each predictor was measured (e.g., instrument, assay, cut-offs); report inter-rater/inter-assay reliability if relevant | Y | Methods (2.3)/SM | Assessed by 2 senior neuroradiologists (blinded to outcomes); Cohen’s kappa=0.82 (NVC grade), ICC=0.89 (quantitative measures); **full inter-reader reliability analysis (SM)** |
| **Methods: Predictors** | 13 | For radiomics/omics features: describe preprocessing, feature extraction, and standardization methods; report reproducibility | Y | Methods (2.3.4)/SM | IBSI-compliant preprocessing; PyRadiomics v3.0.1 for extraction; ICC>0.75 for 92% radiomics features; **radiomics pipeline code (SM)** |
| **Methods: Predictors** | 14 | Describe how missing predictor data were handled (e.g., complete case analysis, imputation; report the proportion of missing data per predictor) | Y | Methods (2.6.1) | Missing data <3% for all predictors; Median imputation (continuous), mode imputation (categorical) |
| **Methods: Outcome(s)** | 15 | Define the primary outcome with explicit criteria; specify the prediction time horizon (i.e., when the outcome is assessed relative to predictor measurement) | Y | Methods (2.5) | Primary outcome: **MVD treatment success** (BNI Grade I/II at final follow-up); Time horizon: ≥6 months post-MVD surgery |
| **Methods: Outcome(s)** | 16 | Describe how the outcome was ascertained (e.g., assessment methods, blinding of assessors); report inter-rater reliability if relevant | Y | Methods (2.5) | Assessed by structured clinical interview; Blinded assessors (not involved in surgery); No inter-rater reliability needed (single assessor per follow-up) |
| **Methods: Model Development** | 17 | Describe all predictor preprocessing steps (e.g., normalization, categorization, one-hot encoding); state if steps were applied within resampling folds to avoid data leakage | Y | Methods (2.6.1) | Z-score normalization (continuous), one-hot encoding (categorical); **all steps performed within CV training folds** (no leakage) |
| **Methods: Model Development** | 18 | Describe the feature selection process (e.g., univariate tests, recursive elimination, regularization); report the final set of predictors included in the model | Y | Methods (2.6.1)/Results (3.4) | 2-stage: Univariate analysis (p<0.05) → RFE-CV; Final predictors: 12 total (TOP5: NVC severity, neurovascular angle, nerve atrophy, arterial compression, proximal NVC) |
| **Methods: Model Development** | 19 | Specify the machine learning (ML) algorithm(s) used (e.g., regression, random forest, XGBoost); justify the choice of algorithm(s) | Y | Methods (2.6.2) | 4 algorithms: Logistic regression (L2), SVM (RBF), random forest, XGBoost; Justification: Proven efficacy for medical prediction tasks (diverse modeling approaches) |
| **Methods: Model Development** | 20 | Describe hyperparameter optimization (e.g., grid search, Bayesian optimization); state if performed within resampling folds; report random seed(s) for reproducibility | Y | Methods (2.6.2)/SM | Grid search + nested CV (within training folds); Random seeds set for all ML models; **all random seeds provided (SM)** |
| **Methods: Model Development** | 21 | Describe methods to address class imbalance (if present; e.g., weighting, oversampling/undersampling, penalization) | Y | Methods (2.6.2) | Class imbalance (93% success/7% failure); Addressed via **penalized regression + class weight adjustment** |
| **Methods: Model Validation** | 22 | Describe the validation approach (e.g., internal: cross-validation; external: independent cohort); specify the type of cross-validation (e.g., k-fold, stratified) | Y | Methods (2.6.2)/SM | **Internal validation: 10-fold stratified cross-validation**; Sensitivity analysis: Repeated 10-fold CV (100 iterations); **repeated CV results (SM)** |
| **Methods: Model Validation** | 23 | Clearly distinguish between apparent performance (on development data) and validated performance (on test data/CV folds); report only validated performance for inference | Y | Methods (2.6.2)/Results (3.3) | All reported performance metrics = **internally validated (CV) results**; no apparent performance reported |
| **Methods: Model Validation** | 24 | Describe methods for model interpretation (e.g., SHAP, LIME, feature importance); clarify limitations (e.g., no causal inference) | Y | Methods (2.6.3)/Discussion (6) | Interpretation via **SHAP values**; Limitations stated: Only minimal interpretability, no causal/actionable clinical insights |
| **Methods: Model Performance Assessment** | 25 | Report discrimination metrics (e.g., AUC-ROC, AUC-PR [for imbalanced data]); provide 95% confidence intervals (CIs) | Y | Results (3.3)/Table 3 | XGBoost: AUC-ROC=0.847 (95%CI:0.752-0.925), **AUC-PR=0.623 (95%CI:0.418-0.812)** (prioritized for imbalance) |
| **Methods: Model Performance Assessment** | 26 | Report calibration metrics (e.g., calibration intercept/slope, Brier score, Hosmer-Lemeshow test); provide 95% CIs; plot a calibration curve | Y | Results (3.3)/Figure 4/Table 3 | XGBoost: Intercept=0.08 (95%CI:-0.05-0.21), Slope=0.94 (95%CI:0.78-1.10), Brier=0.127 (95%CI:0.089-0.176); HL p=0.63; Calibration curve plotted |
| **Methods: Model Performance Assessment** | 27 | Report clinical utility metrics (e.g., decision curve analysis [DCA], clinical impact curve); specify the clinical decision the model supports | Y | Methods (2.6.3)/Results (3.3)/Figure 5 | **DCA** for clinical decision: *Offering MVD vs alternative/deferred treatment* for refractory TN; Net benefit across 15-60% probability thresholds |
| **Methods: Model Performance Assessment** | 28 | Report classification metrics at a prespecified threshold (e.g., Youden’s index, clinical threshold): accuracy, sensitivity, specificity, PPV, NPV; provide a confusion matrix | Y | Results (3.3)/Table 3/SM | Youden’s index threshold; XGBoost: Acc=82.3%, Sen=78.6%, Spe=87.5%, PPV=96.7%, NPV=51.9%; **confusion matrices for all models (SM)** |
| **Methods: Model Performance Assessment** | 29 | Describe subgroup and interaction analyses (e.g., by age, sex, disease severity); state if exploratory/hypothesis-driven; report 95% CIs | Y | Methods (2.7)/Results (3.5)/Table 4 | **Exploratory subgroup analyses** (age, sex, symptom duration, branch involvement); No significant interactions; 95% CIs reported for all metrics |
| **Results** | 30 | Report baseline characteristics of the study population; group by outcome status (event/non-event) and report group comparisons | Y | Results (3.1)/Table 1 | Baseline demographics/clinical features; Stratified by treatment success/failure; Key difference: NVC severity grade (p=0.003) |
| **Results** | 31 | Report univariate associations between each predictor and the outcome (e.g., OR, HR, correlation coefficient; p-values, 95% CIs) | Y | Results (3.2)/Table 2 | Univariate OR (95%CI) and p-values for all MRI predictors; Strongest association: Severe NVC (OR=8.44, 95%CI:1.02-69.8, p=0.003) |
| **Results** | 32 | Report performance metrics for all ML algorithms tested; identify the final/optimal model and justify the choice | Y | Results (3.3)/Table 3/Figure 3 | Performance for all 4 algorithms reported; **XGBoost (gradient boosting)** = optimal (highest AUC-ROC/AUC-PR, best calibration) |
| **Results** | 33 | Report the relative importance of all predictors in the final model; verify consistency with univariate analyses (if relevant) | Y | Results (3.4)/Figure 6/Table 1 | SHAP feature importance ranking; **NVC severity (1st)** = consistent with univariate significance (p=0.003); all top features align with univariate findings |
| **Results** | 34 | Plot the calibration curve for the final model; show the relationship between predicted probabilities and observed outcomes | Y | Results (3.3)/Figure 4 | Calibration curve for XGBoost; Curve closely follows ideal diagonal (excellent calibration) |
| **Results** | 35 | Plot the clinical utility analysis (e.g., DCA); interpret the net benefit and its clinical implications | Y | Results (3.3)/Figure 5 | DCA plot for XGBoost; Positive net benefit vs. treat all/treat none (15-60% thresholds); Clinically meaningful for MVD candidate selection |
| **Discussion** | 36 | Summarize the key findings (e.g., model performance, most important predictors, incremental value over existing tools) | Y | Discussion (1) | XGBoost AUC=0.847; TOP5 MRI predictors; 26% higher AUC-ROC vs. clinical-only baseline (96% higher AUC-PR); significant incremental value |
| **Discussion** | 37 | Compare the study findings with existing literature (e.g., other prediction models for the same outcome; predictor importance) | Y | Discussion (2-5) | Consistent with prior studies (severe NVC/arterial compression = favorable predictors); Novel finding: Neurovascular angle threshold (<45°) for optimal outcomes |
| **Discussion** | 38 | Discuss the study limitations (e.g., study design, sample size, EPP, missing data, no external validation, limited generalizability) | Y | Discussion (8) | Key limitations: Retrospective single-center, small sample size (low EPP), no external validation, manual feature extraction, short follow-up |
| **Discussion** | 39 | Interpret the results in the context of the intended use; explain the potential clinical impact and implementation considerations | Y | Discussion (last)/Conclusion | Clinical impact: Personalized surgical counseling, optimized MVD patient selection; Aligned with ERAS/precision neurosurgery; Implementation: Automation needed for clinical workflow |
| **Discussion** | 40 | Propose specific future research (e.g., external validation in multi-center cohorts, model updating, head-to-head comparison with clinical tools) | Y | Conclusion (last) | Future work: **Multi-center external validation**, automated feature extraction, integrate clinical/functional outcomes, robust explainability frameworks |
| **Other** | 41 | State compliance with the TRIPOD-AI (and TRIPOD 2015) guidelines; provide the completed checklist as supplementary material | Y | Introduction (4)/SM | Explicitly stated compliance; **completed TRIPOD-AI/STROBE checklists (SM)** with page/line mapping |
| **Other** | 42 | Report ethics approval details (e.g., committee name, approval number, date); state if informed consent was obtained/waivers | Y | Methods (2.1)/Manuscript end | IRB approval (Number: [XXX-2020], Date: [XX/XX/2020]); Informed consent **waived** (retrospective study); covers secondary data/imaging use |
| **Other** | 43 | Provide a data and code availability statement; specify how to access the minimal dataset, ML code, and preprocessing scripts | Y | Manuscript end/SM | Minimal dataset: Available from corresponding author (ethics restrictions); **ML/radiomics code + random seeds (SM)** (PyRadiomics v3.0.1, Python 3.9) |
| **Other** | 44 | Discuss the generalizability (external validity) of the model; define the intended population for its use | Y | Discussion (8)/Conclusion | Generalizability limited to **medication-refractory classical TN patients undergoing primary MVD** (tertiary center); Not generalizable to secondary TN/prior surgery patients |
