# Supplementary material for: Retrospective study: preoperative MRI features for predicting microvascular decompression outcomes in trigeminal neuralgia
Source: BMC Med Inform Decis Mak. 2026 Aug 3;26:443. doi: 10.1186/s12911-026-03745-1 (PMC13721266; doi:10.1186/s12911-026-03745-1)
Supplement: Supplementary file 2 [file 12911_2026_3745_MOESM2_ESM.docx]

STROBE Checklist (for Retrospective Cohort Studies)

**Study Title**: Feasibility of Preoperative MRI Features for Predicting Microvascular Decompression Outcomes in Patients with Trigeminal Neuralgia

**Checklist Notes**: Completed in accordance with the STROBE 2015 guidelines (for cohort studies); the corresponding **section/paragraph** in the revised manuscript is marked. **Y=Reported N=Not reported NA=Not applicable**

| **No.** | **Item** | **Report Status** | **Corresponding Manuscript Location** | **Remarks** |
| --- | --- | --- | --- | --- |
| **Title/Abstract** |  |  |  |  |
| 1 | Clearly label the study design as a **retrospective cohort study** in the title/abstract | Y | Title/Abstract: Methods section | Labeled "retrospective single-center observational study" |
| 2 | The abstract includes the core elements of the study: study population, exposure (MRI features), outcome, sample size, main results, conclusion | Y | Whole Abstract | Structured presentation with no missing core elements |
| **Introduction - Research Background** |  |  |  |  |
| 3 | Elaborate on the clinical background of MVD treatment for trigeminal neuralgia and explain the research necessity of preoperative outcome prediction | Y | Introduction: Paragraphs 1-3 | Elaborated in combination with disease burden and existing clinical diagnosis and treatment deficiencies |
| 4 | Clearly state the research purpose and research question, and explain the primary study endpoint (MVD treatment success) | Y | Introduction: Paragraph 4/Abstract: Objective | The research purpose and question correspond clearly |
| **Methods - Study Design** |  |  |  |  |
| 5 | Clearly describe the study design type and explain the study implementation time and location (Jan 2020-Dec 2024, single tertiary neurosurgical center) | Y | Methods: 2.1 Study Design and Participants | Time, location and study design type are all clearly stated |
| 6 | Explain the recruitment method of study subjects (consecutive enrollment) and the time nodes of follow-up start and end | Y | Methods: 2.1 Study Design and Participants/2.5 Outcome Assessment | Consecutively enroll eligible patients; follow-up until ≥6 months postoperatively |
| 7 | Explain the basis for sample size determination and report the EPP ratio and overfitting risk control measures | Y | Methods: 2.1 Study Design and Participants | Based on a feasibility study; supplement EPP and risk control measures |
| **Methods - Study Population** |  |  |  |  |
| 8 | Detailedly list the **inclusion/exclusion criteria** of the study population, and clearly state the diagnostic criteria (ICHD-3) and surgical indications | Y | Methods: 2.1 Study Design and Participants | Specific inclusion and exclusion criteria; clear diagnostic/surgical indications |
| 9 | Report the **study subject screening process**: number of initial screenings, specific number of exclusions for each reason, and final enrolled number | Y | Methods: 2.1 Study Design and Participants | 148 → 48 → 100; report exclusions by reason |
| 10 | Explain the collection method of baseline data of study subjects (review of electronic medical records and imaging data) | Y | Methods: 2.1 Study Design and Participants/2.3 Image Analysis and Feature Extraction | Clear data sources, collected retrospectively |
| **Methods - Exposure (Predictors)** |  |  |  |  |
| 11 | Clearly define the exposure factors (preoperative MRI features) and describe the measurement methods in detail in four categories | Y | Methods: 2.3 Image Analysis and Feature Extraction (2.3.1-2.3.4) | Clear definition of exposure factors; specific measurement methods |
| 12 | Explain the evaluators of exposure factors (2 senior neuroradiologists), assessment blinding (blinded to outcomes), and consistency test | Y | Methods: 2.3 Image Analysis and Feature Extraction | Report kappa and ICC to verify the reliability of assessment |
| 13 | Explain the extraction, preprocessing and standardization methods of radiomics features to ensure the reproducibility of measurements | Y | Methods: 2.3.4 Radiomics Feature Extraction | Implemented in accordance with IBSI guidelines; report reproducibility test results |
| 14 | Explain the missing value ratio and processing method of exposure factors | Y | Methods: 2.6.1 Feature Selection and Preprocessing | Missing value <3%; reasonable processing methods |
| **Methods - Outcome** |  |  |  |  |
| 15 | Clearly define the study outcome (MVD treatment success) and explain the outcome assessment criteria (BNI grade) and time horizon | Y | Methods: 2.5 Outcome Assessment | Unified outcome definition; clear assessment criteria and time horizon |
| 16 | Explain the outcome evaluators (physicians not involved in surgery), assessment method (structured clinical interview), and blinding implementation | Y | Methods: 2.5 Outcome Assessment | Evaluators are independent to reduce outcome assessment bias |
| 17 | Explain the quality control measures for outcome assessment, such as standardized follow-up procedures | Y | Methods: 2.5 Outcome Assessment | Structured interviews with fixed follow-up time points |
| **Methods - Confounders** |  |  |  |  |
| 18 | List all considered confounders (age, sex, symptom duration, branch involvement, preoperative medication, etc.) | Y | Table 1/Methods: 2.7 Statistical Analysis | Comprehensive confounders covering demographic and clinical characteristics |
| 19 | Explain the control methods of confounders in data analysis (multivariate model, subgroup analysis) | Y | Methods: 2.6 Machine Learning Model Development/2.7 Statistical Analysis | Integrate confounders through machine learning models; verify by subgroup analysis |
| **Methods - Statistical Analysis** |  |  |  |  |
| 20 | Explain the descriptive statistical methods for baseline characteristics (mean±SD/median IQR, frequency/ percentage) | Y | Methods: 2.7 Statistical Analysis/Table 1 | Select appropriate methods according to the distribution type of variables |
| 21 | Explain the statistical methods for inter-group comparison (t-test/Mann-Whitney U, chi-square/Fisher's exact test) | Y | Methods: 2.7 Statistical Analysis/Table 1/Table 2 | Select appropriate tests according to variable type and distribution |
| 22 | Explain the construction, validation and performance evaluation methods of machine learning models, and list all reported statistical metrics | Y | Methods: 2.6 Machine Learning Model Development/2.7 Statistical Analysis | Complete model process and comprehensive performance metrics |
| 23 | Explain the methods of subgroup analysis and interaction analysis, and clearly position them as exploratory analyses | Y | Methods: 2.7 Statistical Analysis/Results: 3.5 Subgroup Analyses | Clear subgroup analysis methods; labeled as exploratory |
| 24 | Explain the significance level of statistical tests (α=0.05); report 95%CI for all interval estimates | Y | Methods: 2.7 Statistical Analysis | Unified significance level throughout the manuscript; complete interval estimates |
| 25 | Explain the software used for statistical analysis (Python 3.9, scikit-learn/pandas/scipy) | Y | Methods: 2.7 Statistical Analysis | Clear software and version to ensure reproducibility |
| **Results - Study Population** |  |  |  |  |
| 26 | Report the baseline demographic and clinical characteristics of the study population, display by outcome group and report inter-group comparison results | Y | Results: 3.1 Baseline Characteristics and Surgical Outcomes/Table 1 | Complete baseline characteristics; significant inter-group differences in key features |
| 27 | Report the follow-up completion of the study population, including follow-up duration, number of lost to follow-up and reasons for loss to follow-up | Y | Methods: 2.1 Study Design and Participants/Results: 3.1 Baseline Characteristics and Surgical Outcomes | 1 patient lost to follow-up with clear reasons; 99% follow-up completion rate |
| 28 | Report the distribution of study subjects stratified by exposure factors (e.g., distribution of NVC grade and compression type) | Y | Results: 3.1 Baseline Characteristics and Surgical Outcomes/Table 2 | Clear distribution of exposure factors with definite correlation with outcomes |
| **Results - Association between Exposure and Outcome** |  |  |  |  |
| 29 | Report the univariate association analysis results of each exposure factor with the outcome (p-value, OR/95%CI) | Y | Results: 3.2 Imaging Features Associated with Treatment Outcomes/Table 2 | Comprehensive univariate analysis results to quantify the strength of association |
| 30 | Report the performance results of machine learning models and clarify the core metrics and feature importance of the optimal model | Y | Results: 3.3 Machine Learning Model Performance/3.4 Feature Importance Analysis/Table 3/Figure 6 | Outstanding model performance with clear feature importance ranking |
| 31 | Report the subgroup analysis results and explain the performance stability of the model in different subgroups | Y | Results: 3.5 Subgroup Analyses/Table 4 | Robust subgroup performance with no significant interaction |
| 32 | Report the clinical utility analysis results of the model (DCA) and explain its net benefit and clinical application value | Y | Results: 3.3 Machine Learning Model Performance/Figure 5 | Clear DCA results with significant clinical utility |
| **Discussion** |  |  |  |  |
| 33 | Summarize the main results of the study and clearly answer the research question raised | Y | Discussion: Paragraph 1 | Core results correspond to the research question with a clear conclusion |
| 34 | Compare the results of this study with existing relevant studies and analyze the reasons for consistency and difference | Y | Discussion: Paragraphs 2-5 | Combine previous literature to analyze result differences in depth |
| 35 | Clearly elaborate on the **limitations** of the study, including methodological and clinical deficiencies | Y | Discussion: Paragraph 8 | Comprehensive and objective elaboration of limitations without avoidance |
| 36 | Combine the study limitations and existing evidence to explain the **generalizability** of the study results (applicable population) | Y | Discussion: Paragraph 8/Conclusion | Clearly define the applicable boundary of the model to avoid over-generalization |
| 37 | Explain the **clinical significance** of the study results and propose implications for clinical practice and future research | Y | Discussion: Last paragraph/Conclusion | Clear clinical significance and specific future research directions |
| **Other** |  |  |  |  |
| 38 | State the ethics approval of the study, including approval number, date, and reasons for waiving informed consent | Y | Methods: 2.1 Study Design and Participants/Ethics Approval Statement at the end of the manuscript | Completed in accordance with BMC/ICMJE standards |
| 39 | Explain the funding source and conflict of interest of the study | NA | At the end of the manuscript | No funding source and no conflict of interest for this study |
| 40 | Provide a data and code availability statement and explain the reproducibility of the study | Y | Data and Code Availability Statement at the end of the manuscript | Compliant with BMC journal requirements; provide access methods |
| 41 | State compliance with STROBE/TRIPOD-AI guidelines and provide complete checklists | Y | Introduction: Paragraph 4/Supplementary Materials Note | Clearly stated; checklists are submitted as supplementary materials |
